# The *CHEK2* Variant C.349A>G Is Associated with Prostate Cancer Risk and Carriers Share a Common Ancestor

**DOI:** 10.3390/cancers12113254

**Published:** 2020-11-04

**Authors:** Andreia Brandão, Paula Paulo, Sofia Maia, Manuela Pinheiro, Ana Peixoto, Marta Cardoso, Maria P. Silva, Catarina Santos, Rosalind A. Eeles, Zsofia Kote-Jarai, Kenneth Muir, UKGPCS Collaborators, Johanna Schleutker, Ying Wang, Nora Pashayan, Jyotsna Batra, APCB BioResource, Henrik Grönberg, David E. Neal, Børge G. Nordestgaard, Catherine M. Tangen, Melissa C. Southey, Alicja Wolk, Demetrius Albanes, Christopher A. Haiman, Ruth C. Travis, Janet L. Stanford, Lorelei A. Mucci, Catharine M. L. West, Sune F. Nielsen, Adam S. Kibel, Olivier Cussenot, Sonja I. Berndt, Stella Koutros, Karina Dalsgaard Sørensen, Cezary Cybulski, Eli Marie Grindedal, Jong Y. Park, Sue A. Ingles, Christiane Maier, Robert J. Hamilton, Barry S. Rosenstein, Ana Vega, The IMPACT Study Steering Committee and Collaborators, Manolis Kogevinas, Fredrik Wiklund, Kathryn L. Penney, Hermann Brenner, Esther M. John, Radka Kaneva, Christopher J. Logothetis, Susan L. Neuhausen, Kim De Ruyck, Azad Razack, Lisa F. Newcomb, Canary PASS Investigators, Davor Lessel, Nawaid Usmani, Frank Claessens, Manuela Gago-Dominguez, Paul A. Townsend, Monique J. Roobol, The Profile Study Steering Committee, The PRACTICAL Consortium, Manuel R. Teixeira

**Affiliations:** 1 Cancer Genetics Group, IPO Porto Research Center (CI-IPOP), Portuguese Oncology Institute of Porto (IPO Porto), 4200-072 Porto, Portugal; andreia.aguiar.brandao@ipoporto.min-saude.pt (A.B.); paula.paulo@ipoporto.min-saude.pt (P.P.); s.maia@chuc.min-saude.pt (S.M.); manuelap@ipoporto.min-saude.pt (M.P.); marta.jose.cardoso@ipoporto.min-saude.pt (M.C.); maria.pereira.silva@ipoporto.min-saude.pt (M.P.S.); 2 Department of Genetics, Portuguese Oncology Institute of Porto (IPO Porto), 4200-072 Porto, Portugal; analuisamoura@ipoporto.min-saude.pt (A.P.); catarinasantos@ipoporto.min-saude.pt (C.S.); 3 The Institute of Cancer Research, London SM2 5NG, UK; Ros.Eeles@icr.ac.uk (R.A.E.); ZSofia.Kote-Jarai@icr.ac.uk (Z.K.-J.); 4 Royal Marsden NHS Foundation Trust, London SW3 6JJ, UK; 5 Division of Population Health, Health Services Research and Primary Care, University of Manchester, Oxford Road, Manchester M13 9PL, UK; kenneth.muir@manchester.ac.uk; 6 Warwick Medical School, University of Warwick, Coventry CV4 7AL, UK; 7 The Institute of Cancer Research, London SW7 3RP, UK; ukgpcs@icr.ac.uk (UKGPCS Collaborators); impact-study@icr.ac.uk (The IMPACT Study Steering Committee and Collaborators); 8 Institute of Biomedicine, University of Turku, FI-20014 Turun Yliopisto, 20050 Turku, Finland; Johanna.Schleutker@utu.fi; 9 Department of Medical Genetics, Genomics, Laboratory Division, Turku University Hospital, P.O. Box 52, 20521 Turku, Finland; 10 Department of Population Science, American Cancer Society, 250 Williams Street, Atlanta, GA 30303, USA; ying.wang@cancer.org; 11 Department of Applied Health Research, University College London, London WC1E 7HB, UK; n.pashayan@ucl.ac.uk; 12 Centre for Cancer Genetic Epidemiology, Department of Oncology, University of Cambridge, Strangeways Research Laboratory, Worts Causeway, Cambridge CB1 8RN, UK; 13 Australian Prostate Cancer Research Centre-Qld, Institute of Health and Biomedical Innovation and School of Biomedical Sciences, Queensland University of Technology, Brisbane, QLD 4059, Australia; jyotsna.batra@qut.edu.au (J.B.); j.clements@qut.edu.au (APCB BioResource); 14 Translational Research Institute, Brisbane, QLD 4102, Australia; 15 Department of Medical Epidemiology and Biostatistics, Karolinska Institute, SE-171 77 Stockholm, Sweden; henrik.gronberg@ki.se (H.G.); Fredrik.Wiklund@ki.se (F.W.); 16 Nuffield Department of Surgical Sciences, University of Oxford, Room 6603, Level 6, John Radcliffe Hospital, Headley Way, Headington, Oxford OX3 9DU, UK; den22@medschl.cam.ac.uk; 17 Department of Oncology, University of Cambridge, Addenbrooke’s Hospital, Hills Road, Cambridge CB2 0QQ, UK; 18 Cancer Research UK, Cambridge Research Institute, Li Ka Shing Centre, Cambridge CB2 0RE, UK; 19 Faculty of Health and Medical Sciences, University of Copenhagen, 2200 Copenhagen, Denmark; Boerge.Nordestgaard@regionh.dk (B.G.N.); Sune.Fallgaard.Nielsen@regionh.dk (S.F.N.); 20 Department of Clinical Biochemistry, Herlev and Gentofte Hospital, Copenhagen University Hospital, Herlev, 2200 Copenhagen, Denmark; 21 SWOG Statistical Center, Fred Hutchinson Cancer Research Center, 1100 Fairview Avenue North, M3-C102, Seattle, WA 98109-1024, USA; ctangen@fredhutch.org; 22 Precision Medicine, School of Clinical Sciences at Monash Health, Monash University, Clayton, VIC 3168, Australia; melissa.southey@monash.edu; 23 Cancer Epidemiology Division, Cancer Council Victoria, 615 St Kilda Road, Melbourne, VIC 3004, Australia; 24 Department of Clinical Pathology, The Melbourne Medical School, The University of Melbourne, Melbourne, VIC 3004, Australia; 25 Unit of Cardiovascular and Nutritional Epidemiology, Institute of Environmental Medicine, Karolinska Institutet, SE-171 77 Stockholm, Sweden; Alicja.Wolk@ki.se; 26 Department of Surgical Sciences, Uppsala University, 75185 Uppsala, Sweden; 27 Division of Cancer Epidemiology and Genetics, National Cancer Institute, NIH, Bethesda, ML 20892, USA; DAA@NIH.GOV (D.A.); berndts@mail.nih.gov (S.I.B.); koutross@mail.nih.gov (S.K.); 28 Center for Genetic Epidemiology, Department of Preventive Medicine, Keck School of Medicine, University of Southern California/Norris Comprehensive Cancer Center, Los Angeles, CA 90015, USA; Christopher.Haiman@med.usc.edu; 29 Cancer Epidemiology Unit, Nuffield Department of Population Health, University of Oxford, Oxford OX3 7LF, UK; ruth.travis@ceu.ox.ac.uk; 30 Division of Public Health Sciences, Fred Hutchinson Cancer Research Center, Seattle, DC 98109-1024, USA; jstanfor@fhcrc.org (J.L.S.); lnewcomb@fredhutch.org (L.F.N.); dlin@uw.edu (Canary PASS Investigators); 31 Department of Epidemiology, School of Public Health, University of Washington, Seattle, DC 98195, USA; 32 Department of Epidemiology, Harvard T. H. Chan School of Public Health, Boston, MA 02115, USA; lmucci@hsph.harvard.edu; 33 Division of Cancer Sciences, University of Manchester, Manchester Academic Health Science Centre, Radiotherapy Related Research, The Christie Hospital NHS Foundation Trust, Manchester M13 9PL, UK; catharine.west@manchester.ac.uk; 34 Division of Urologic Surgery, Brigham and Womens Hospital, 75 Francis Street, Boston, MA 02115, USA; akibel@bwh.harvard.edu; 35 Sorbonne Universite, GRC n 5, AP-HP, Tenon Hospital, 4 rue de la Chine, F-75020 Paris, France; olivier.cussenot@aphp.fr; 36 CeRePP, Tenon Hospital, F-75020 Paris, France; 37 Department of Molecular Medicine, Aarhus University Hospital, Palle Juul-Jensen Boulevard 99, 8200 Aarhus N, Denmark; kdso@clin.au.dk; 38 Department of Clinical Medicine, Aarhus University, DK-8200 Aarhus N, Denmark; 39 International Hereditary Cancer Center, Department of Genetics and Pathology, Pomeranian Medical University, 70-115 Szczecin, Poland; cezarycy@pum.edu.pl; 40 Department of Medical Genetics, Oslo University Hospital, 0424 Oslo, Norway; ELIGR@ous-hf.no; 41 Department of Cancer Epidemiology, Moffitt Cancer Center, 12902 Magnolia Drive, Tampa, FL 33612, USA; Jong.Park@moffitt.org; 42 Department of Preventive Medicine, Keck School of Medicine, University of Southern California/Norris Comprehensive Cancer Center, Los Angeles, CA 90015, USA; ingles@usc.edu; 43 Humangenetik Tuebingen, Paul-Ehrlich-Str 23, D-72076 Tuebingen, Germany; christiane.maier@humangenetik-tuebingen.de; 44 Department of Surgical Oncology, Princess Margaret Cancer Centre, Toronto, ON M5G 2M9, Canada; rob.hamilton@uhn.ca; 45 Department of Surgery (Urology), University of Toronto, Toronto, ON M5T 1P5, Canada; 46 Department of Radiation Oncology and Department of Genetics and Genomic Sciences, Box 1236, Icahn School of Medicine at Mount Sinai, One Gustave L. Levy Place, New York, NY 10029, USA; barry.rosenstein@mssm.edu; 47 Department of Genetics and Genomic Sciences, Icahn School of Medicine at Mount Sinai, New York, NY 10029-5674, USA; 48 Fundación Pública Galega Medicina Xenómica, 15706 Santiago de Compostela, Spain; ana.vega@usc.es; 49 Health Research Institute of Santiago de Compostela (IDIS), 15706 Santiago De Compostela, Spain; 50 CIBER of Rare Diseases (CIBERER), 28029 Madrid, Spain; 51 ISGlobal, 08036 Barcelona, Spain; manolis.kogevinas@isglobal.org; 52 IMIM (Hospital del Mar Medical Research Institute), 08003 Barcelona, Spain; 53 Campus del Mar, Universitat Pompeu Fabra (UPF), 08003 Barcelona, Spain; 54 CIBER Epidemiología y Salud Pública (CIBERESP), 28029 Madrid, Spain; 55 Channing Division of Network Medicine, Department of Medicine, Brigham and Women’s Hospital/Harvard Medical School, Boston, MA 02184, USA; kpenney@hsph.harvard.edu; 56 Division of Clinical Epidemiology and Aging Research, German Cancer Research Center (DKFZ), 69120 Heidelberg, Germany; h.brenner@dkfz.de; 57 German Cancer Consortium (DKTK), German Cancer Research Center (DKFZ), D-69120 Heidelberg, Germany; 58 Division of Preventive Oncology, German Cancer Research Center (DKFZ) and National Center for Tumor Diseases (NCT), Im Neuenheimer Feld 460, 69120 Heidelberg, Germany; 59 Departments of Epidemiology & Population Health and of Medicine, Division of Oncology, Stanford Cancer Institute, Stanford University School of Medicine, Stanford, CA 94304, USA; emjohn@stanford.edu; 60 Molecular Medicine Center, Department of Medical Chemistry and Biochemistry, Medical University of Sofia, Sofia, 2 Zdrave Str., 1431 Sofia, Bulgaria; kaneva@mmcbg.org; 61 Department of Genitourinary Medical Oncology, The University of Texas M. D. Anderson Cancer Center, 1515 Holcombe Blvd., Houston, TX 77030, USA; clogothe@mdanderson.org; 62 Department of Population Sciences, Beckman Research Institute of the City of Hope, 1500 East Duarte Road, Duarte, CA 91010, USA; sneuhausen@coh.org; 63 Faculty of Medicine and Health Sciences, Basic Medical Sciences, Ghent University, Proeftuinstraat 86, 9000 Gent, Belgium; Kim.DeRuyck@ugent.be; 64 Department of Surgery, Faculty of Medicine, University of Malaya, 50603 Kuala Lumpur, Malaysia; azad@um.edu.my; 65 Department of Urology, University of Washington, 1959 NE Pacific Street, Box 356510, Seattle, WA 98195, USA; 66 Institute of Human Genetics, University Medical Center Hamburg-Eppendorf, 20246 Hamburg, Germany; d.lessel@uke.de; 67 Department of Oncology, Cross Cancer Institute, University of Alberta, 11560 University Avenue, Edmonton, AB T6G 1Z2, Canada; Nawaid.Usmani@albertahealthservices.ca; 68 Division of Radiation Oncology, Cross Cancer Institute, 11560 University Avenue, Edmonton, AB T6G 1Z2, Canada; 69 Molecular Endocrinology Laboratory, Department of Cellular and Molecular Medicine, Campus Gasthuisberg, University of Leuven, Herestraat 49, P.O. Box 901, 3000 Leuven, Belgium; frank.claessens@med.kuleuven.be; 70 Group of Genomic Medicine, Galician Public Foundation of Genomic Medicine, Health Research Institute of Santiago de Compostela (IDIS), Galician Healthcare Service (SERGAS) University of Santiago de Compostela, 15782 Santiago de Compostela, Spain; mgago@med.usc.edu; 71 Moores Cancer Center, Department of Family Medicine and Public Health, University of California San Diego, La Jolla, CA 92093-0012, USA; 72 Division of Cancer Sciences, Manchester Cancer Research Centre, Faculty of Biology, Medicine and Health, Manchester Academic Health Science Centre, National Institute for Health Research (NIHR) Manchester Biomedical Research Centre, Health Innovation Manchester, University of Manchester, Manchester M13 9PL, UK; paul.townsend@manchester.ac.uk; 73 Department of Urology, Erasmus University Medical Center, 3015 CE Rotterdam, The Netherlands; m.roobol@erasmusmc.nl; 74 Institute of Cancer Research and Royal Marsden Hospital, Sutton, Surrey SM2 5PT, UK; elizabeth.bancroft@rmh.nhs.uk; 75 Biomedical Sciences Institute Abel Salazar (ICBAS), University of Porto, 4050-313 Porto, Portugal

**Keywords:** prostate cancer, founder variant, CHEK2, cancer predisposition

## Abstract

**Simple Summary:**

It is well-recognised the strong contribution of genetic factors to prostate cancer (PrCa) susceptibility, thus genetic screening is critical for presymptomatic diagnosis and identification of individuals at high-risk. In this context, recurrent founder variants in cancer predisposing genes, by providing specific targets for early identification of carriers at risk of developing the disease, may be leveraged to implement cost-efficient targeted genetic screening strategies. The goal of this study was to investigate whether *CHEK2* c.349A>G, the only recurrent “likely pathogenic” variant in *CHEK2* gene reported in the Portuguese population, plays an important role in PrCa development, and the possibility of a founder effect behind its origin. Our results clearly demonstrate that c.349A>G in the *CHEK2* tumour-suppressor gene is a founder variant significantly associated with an increased risk of PrCa, suggesting its potential usefulness for cost-effective targeted genetic screening in PrCa families.

**Abstract:**

The identification of recurrent founder variants in cancer predisposing genes may have important implications for implementing cost-effective targeted genetic screening strategies. In this study, we evaluated the prevalence and relative risk of the *CHEK2* recurrent variant c.349A>G in a series of 462 Portuguese patients with early-onset and/or familial/hereditary prostate cancer (PrCa), as well as in the large multicentre PRACTICAL case–control study comprising 55,162 prostate cancer cases and 36,147 controls. Additionally, we investigated the potential shared ancestry of the carriers by performing identity-by-descent, haplotype and age estimation analyses using high-density SNP data from 70 variant carriers belonging to 11 different populations included in the PRACTICAL consortium. The *CHEK2* missense variant c.349A>G was found significantly associated with an increased risk for PrCa (OR 1.9; 95% CI: 1.1–3.2). A shared haplotype flanking the variant in all carriers was identified, strongly suggesting a common founder of European origin. Additionally, using two independent statistical algorithms, implemented by DMLE+2.3 and ESTIAGE, we were able to estimate the age of the variant between 2300 and 3125 years. By extending the haplotype analysis to 14 additional carrier families, a shared core haplotype was revealed among all carriers matching the conserved region previously identified in the high-density SNP analysis. These findings are consistent with *CHEK2* c.349A>G being a founder variant associated with increased PrCa risk, suggesting its potential usefulness for cost-effective targeted genetic screening in PrCa families.

## 1. Introduction

Prostate cancer (PrCa) is one of the most commonly diagnosed cancers worldwide, representing the second leading cause of cancer mortality among men in the developed countries [1]. Despite the strong epidemiological evidence supporting a genetic contribution to PrCa, with 10–20% of the cases expected to occur in a hereditary/familial context, the genetic aetiology is still largely unknown [2]. To date, numerous family-based linkage and genome-wide association studies (GWAS) have reported more than 100 common low-penetrance genetic variants associated with PrCa risk, most of which were identified in populations of European ancestry [3,4,5,6,7,8]. However, no specific high-risk gene for PrCa has been identified. Apart from some well-established moderate-risk genes [9,10,11,12,13,14,15,16,17], a few additional candidate genes have, more recently, been proposed to explain PrCa heritability.

*CHEK2* is a tumour suppressor gene that encodes a serine threonine kinase involved in pathways such as DNA repair, cell cycle arrest, mitosis, and apoptosis [18,19,20]. Although several germline variants in the *CHEK2* gene have been associated with increased cancer risk, the knowledge regarding the full mutational spectra and specific variant-associated risk, particularity in PrCa, is still limited [21]. So far, the c.1100delC and p.I157T *CHEK2* variants are the most comprehensively studied, being associated in large case–control studies with increased risk for different types of cancer, such as testicular germ cell tumours, breast and colorectal cancers [22,23,24]. Other cancer risk-associated *CHEK2* variants have been reported [22], some of which in ethnically defined groups such as the Ashkenazi Jewish population [25], suggesting the influence of founder effects underlying *CHEK2* mutational spectra.

Recently, we performed a comprehensive genetic screening of 94 genes associated with inherited cancer predisposition in a selected series of 121 Portuguese patients with early-onset disease and/or criteria for familial/hereditary PrCa [26]. Only one recurrent variant, namely *CHEK2* c.349A>G, was identified in two Portuguese PrCa families. This variant, classified as “pathogenic/likely pathogenic” by ClinVar (http://www.ncbi.nlm.nih.gov/clinvar/, accessed in January 2020), has already been implicated in previous large-scale studies with increased risk for breast cancer (BrCa), but not PrCa development [22]. The existence of a recurrent “likely pathogenic” variant in the *CHEK2* gene may be the reflection of a founder event. The identification of founder variants in cancer predisposing genes is important to improve risk assessment in specific populations, allowing more cost-efficient screening strategies by providing specific targets for early identification of carriers at risk to develop the disease. It remains unknown whether the *CHEK2* variant c.349A>G may have arisen from a common founder ancestor or independently through time.

In this work, we aimed to further explore the relevance of the *CHEK2* variant c.349A>G in early-onset/familial PrCa, by evaluating its prevalence in a series of 462 Portuguese PrCa patients with early-onset disease and/or criteria for familial/hereditary PrCa [27]. Additionally, we aimed to explore the hypothesis of a possible founder effect in the origin of this *CHEK2* variant by performing haplotype and age estimation analyses in PrCa patients and controls from 11 different populations included in the PRATICAL (Prostate Cancer Association Group to Investigate Cancer Associated Alterations in the Genome) consortium.

## 2. Results

### 2.1. Frequency of the CHEK2 Variant c.349A>G 

To evaluate the previously suggested contribution of *CHEK2* variant c.349A>G to early-onset and/ or familial PrCa risk, we screened a series of 462 early-onset/familial PrCa cases and compared the frequency in cases with that previously obtained for 710 controls. In addition to the two PrCa cases previously reported [26], the c.349A>G variant was found in three PrCa cases, corroborating a higher frequency in cases (*n* = 5) comparing with controls (*n* = 1), rendering a borderline association with increased risk of PrCa (OR 7.7; 95% CI: 0.9–66.6; *p* = 0.06). 

To further investigate the possible association with increased PrCa risk, we increased the statistical power by evaluating the frequency of the *CHEK2* variant c.349A>G among the 91,309 individuals available from the PRATICAL consortium. The variant was found in 52 PrCa cases (including the four of the five patients previously identified in the Portuguese early-onset and/or hereditary PrCa series) and 18 heathy controls belonging to 11 worldwide spread populations of European ancestry (Table S1), corroborating the association with PrCa (OR 1.9; 95% CI: 1.1–3.2; *p* = 0.04) hinted by the analysis of the Portuguese early-onset/familial PrCa series.

### 2.2. Identification of IBD Haplotype and Phylogeographic Analysis

**Figure 1:**
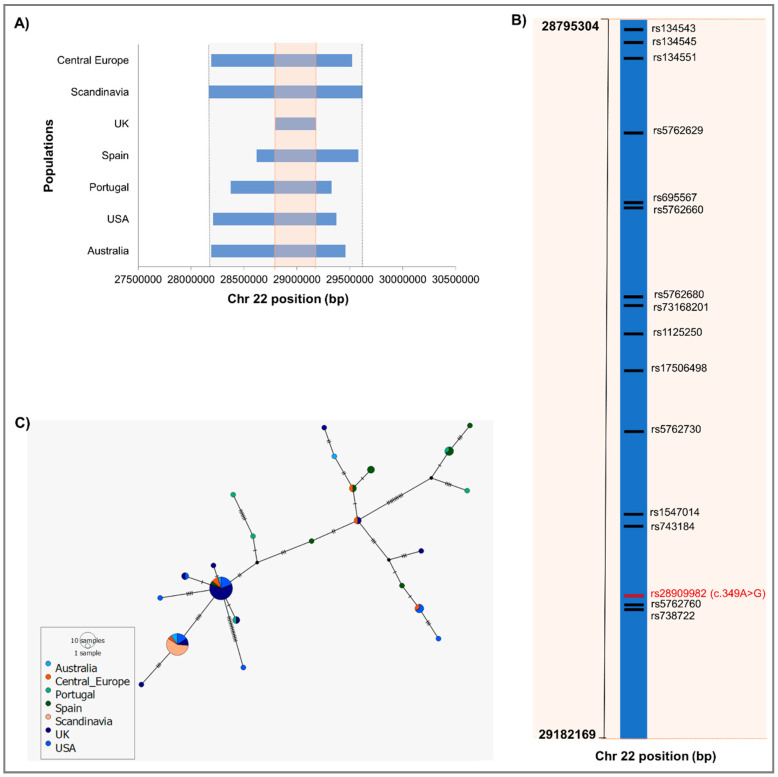
Shared identity-by-descent (IBD) haplotype between all carriers of the *CHEK2* variant c.349A>G. (**A**). Shared IBD segments by populations: Australia, Portugal, Scandinavia (Denmark and Sweden), Spain, Western/Central Europe populations (Belgium, France, Germany and Netherlands), UK, and U.S. (**B**) Characterisation of IBD core segment shared by all carriers (≈0.4 Mb in detail, represented in light orange in Figure 1A). (**C**) Median joining phylogenetic tree of the largest shared haplotype region flanking the *CHEK2* variant c.349A>G between all carriers (indicated in light grey in Figure 1A).

The identity-by-descent (IBD) analysis of the high-density SNP data from chromosome 22 revealed the existence of a shared haplotype with different lengths flanking the *CHEK2* variant c.349A>G among all the 70 variant carriers from the different populations. As presented in Figure 1A, a conserved variant haplotype of ≈1 Mb (chr22: 28,374,461–29,327,347) was found in most of the populations. Noteworthy, we grouped the carriers from France, Germany, Netherlands, and Belgium into a single Western/Central European group, as well as the ones from Denmark and Sweden into a Scandinavian group, due to population size limitations. Interestingly, the Scandinavian carriers revealed the largest conserved haplotype, whereas the UK carriers presented a considerably smaller core haplotype (≈0.4 Mb, chr22: 28,795,304–29,182,169), compared to the other populations. This smaller haplotype consists of 15 common SNPs featured in the OncoArray DNA chip, in addition to the rare *CHEK2* variant c.349A>G (Figure 1B).

The median-joining phylogenetic tree of the largest identified haplotype (≈1.5 Mb, chr22: 28,170,166–29,620,564) flanking the *CHEK2* variant c.349A>G was also consistent with the IBD analysis, and revealed the existence of two major haplotypes shared by most of the populations (Figure 1C).

### 2.3. Age Estimation of the CHEK2 Variant c.349A>G

As the haplotype analysis suggested a founder ancestor among the carriers of the *CHEK2* variant c.349A>G, we sought to estimate its age to obtain further insights regarding its origin and dissemination. A summary of these results is shown in Tables S2 and S3.

**Figure 2:**
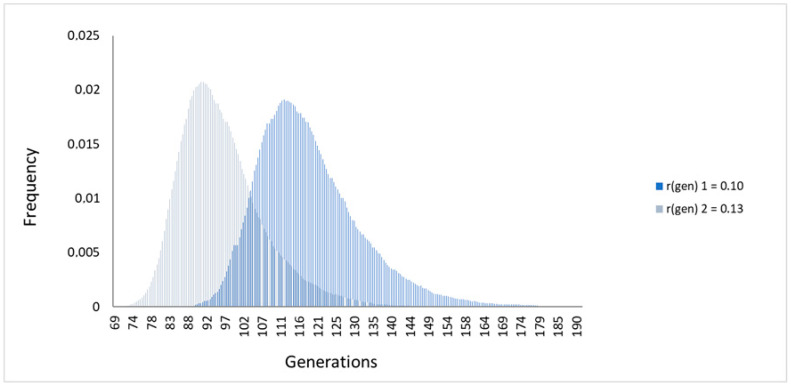
Overall age estimation of the *CHEK2* variant c.349A>G using the DMLE+2.3 software, considering the 70 carriers. Distribution of the posterior probability for the age estimation, assuming 0.00028 as the proportion of variant-carrying chromosomes and the two population growth rates 0.10 and 0.13.

According to DMLE+2.3, the common ancestor of all carriers of the variant originated between 92 (95% CI: 78–118) and 113 (95% CI: 97–145) generations ago, which, considering generations of 25 years, translates into between 2300 and 2825 years ago (Figure 2, Table S2). For comparison, we used a different statistical approach based on a maximum likelihood algorithm employed by ESTIAGE. This analysis provided slightly older range estimates, suggesting that the variant arose approximately 123 (95% CI: 104–146) to 125 (95% CI: 106–148) generations ago, that is, 3075–3125 years ago assuming the 25-year generation time (Table S2).

To enlighten the dispersal patterns of the *CHEK2* variant c.349A>G, we also obtained age estimates for the different populations separately, using DMLE+2.3 (Table S3, Figures S1–S7). However, due to sample size limitations, and to be consistent with the haplotype and phylogeographic analysis, we estimated the variant age in the Western/Central European populations as a group (Belgium, France, Germany and Netherlands), as well in the Scandinavian populations (Denmark and Sweden).

According to the results, the variant appears to have arisen first in the Western/Central region, between 81 (95% CI: 65–104) and 100 (95% CI: 81–132) generations ago, that is, between 2015 and 2500 years ago, assuming a 25-year generation time. Later, dispersed through Spain and Portugal around 61–73 and 53–61 generations ago, respectively, corresponding to the variant dating approximately 1525–1825 years in Spain and 1325–1525 years in Portugal. Slightly younger age estimates were obtained for the UK and Scandinavian carriers, suggesting a common ancestor dating 33 (95% CI: 27–44) to 54 (95% CI: 43–71) generations ago, i.e., 825–1350 years ago, for the first, and dating 42 (95% CI: 31–59) to 49 (95% CI: 38–70) generations ago for the latter, which equates roughly to 1050–1225 years ago.

**Figure 3:**
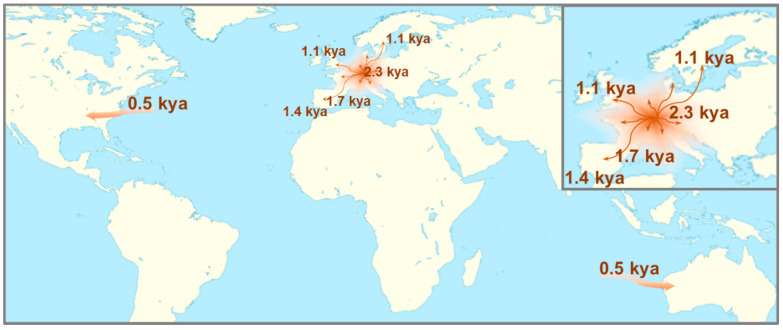
Possible geographic dispersal scenario inferred from present data of the populations carrying *CHEK2* variant c.349A>G, with the average age estimates obtained by DMLE+2.3, using the two population growth rates. Background map adapted from the map outline published under the terms of the GNU Free Documentation License, Version 1.2 [28].

Lastly, DMLE+2.3 estimates suggest that the *CHEK2* variant c.349A>G was introduced more recently to the Australian and U.S. populations. The results revealed a common ancestor dating between 18 (95% CI: 13–24) and 27 (95% CI: 21–38) generations ago among the Australian carriers and dating between 16 (14–21) and 23 (20–29) generations ago among the US. carriers. Therefore, the common ancestor of those two populations arose approximately between 450 and 675 years ago, and between 400 and 575 years ago, respectively (Figure 3).

### 2.4. Haplotype Analysis Using Microsatellites

To further extend the haplotype analysis to additional variant carriers from IPO-Porto, five informative microsatellites markers were analysed on 14 probands with history of prostate, breast, gastric, and lung cancer, and on the 18 additional family members available.

Consistent with the high-density SNPs haplotype findings, the microsatellite analysis also identified a common haplotype of different lengths among all carriers of the variant (Table 1). All five informative families with the c.349A>G variant shared a common haplotype between markers D22S689 and D22S275, spanning a conserved region of approximately of ≈282 Kb. The same haplotype was compatible with the observed genotypes of the nine remaining probands for which the haplotype phase could not be explored, due to the lack of additional family members. The existence of a core haplotype shared among all carriers of the variant, independently of the cancer type, matching the conserved region previously identified in the high-density SNP analysis, strongly corroborates a founder effect in the *CHEK2* variant c.349A>G.

## 3. Discussion

The *CHEK2* gene plays a key role in DNA damage response [18], and although several germline variants have been associated with increased cancer risk, particularly in breast cancer [22,29], the mutational spectra, as observed in other cancer risk genes (e.g., *BRCA1* and *BRCA2* genes [30,31]), varies widely among different populations. For instance, the *CHEK2* variant c.1100delC, which has been shown to increase breast cancer risk by 2-fold [29,32], is frequently found in northern European populations, but is rare in southern European populations [33].

The *CHEK2* variant c.349A>G was initially reported in two *BRCA1/2*-negative familial BrCa patients, but no clear association was found with the disease at the time [34]. More recently, in a large-scale case–control study this variant was associated with an increased risk of BrCa (OR 2.26), but not PrCa [22]. The pathogenic nature of the *CHEK2* variant c.349A>G has been supported by both functional and bioinformatic approaches, which suggested that this variant affects the forkhead-associated (FHA) domain of *CHEK2*, resulting in lack of phosphorylation and oligomerisation, leading to reduced *CHEK2* kinase activity and, ultimately, loss of DNA damage response [26,35,36,37].

To further increase our understanding of the contribution of the *CHEK2* variant c.349A>G, which is, to date, the only recurrent “likely pathogenic” variant in *CHEK2* gene reported in the Portuguese population [26], we completed the genotyping of a series of 462 cases with criteria for early-onset and/or hereditary PrCa. The *CHEK2* variant c.349A>G was more frequent in Portuguese PrCa patients (*n* = 5) compared to controls (*n* = 1), with an odds-ratio suggesting a borderline association with the disease. We sought to validate these findings with the large multicentre case–control PRACTICAL consortium, which comprised 55,162 PrCa cases and 36,147 controls from 53 worldwide studies. The *CHEK2* variant c.349A>G was found in 52 PrCa cases and 18 controls of the PRACTICAL study, providing clear evidence of its association with increased risk for PrCa. A 2- to 3-fold increased PrCa risk has also been linked with other two well-studied *CHEK2* founder variants, p.I157T and c.1100delC, reinforcing the importance of *CHEK2* as a moderate-penetrance PrCa susceptibility gene [21]. A similar modest increased risk has also been reported for men harbouring pathogenic variants in other moderate-penetrance cancer genes, such as *ATM* [38], while a higher risk (up to 8-fold) is described for carriers of alterations in high-penetrance cancer-predisposing genes, such as those associated with hereditary breast and ovarian cancer syndrome (e.g., *BRCA1* and, particularly, *BRCA2*) [39,40]. Noteworthy, a few founder variants prevalent in more genetically homogenous populations, such as the *HOXB13* G84E variant in Nordic populations, have been strongly associated with high risk (OR, 3.4) of PrCa [41].

Another key aspect revealed by these results is the widespread distribution of this variant, which was found in carriers from 11 different countries, namely Australia, Belgium, Denmark, France, Germany, Netherlands, Portugal, Spain, Sweden, the UK, and U.S. The recurrence of the *CHEK2* variant c.349A>G in, apparently, unrelated carriers from diverse populations could be due to independent origin or carriers might share a common ancestor. We addressed this question by performing haplotype analysis using high-density SNP data for all carriers available from the PRACTICAL dataset. The conserved IBD haplotype flanking the *CHEK2* variant c.349A>G in all carriers highly indicates a single common founder. Moreover, the haplotype reconstructed network, characterised with two major haplotypes shared by distinct populations, suggests early recombination events splitting the initial haplotype into distinct haplotypes in the founding population, most likely of Central European origin, which were then carried as it rapidly spread. Noteworthy, a large conserved haplotype was identified among all Scandinavian carriers, suggesting some degree of isolation after the introduction of the variant into the population, whereas the UK carriers presented the smallest conserved haplotype, most likely as result of this population history of extensive migration waves introducing distinct levels of genetic differentiation into the region [42].

The conserved haplotype allied with the age estimates obtained by the two independent mathematical approaches, the Bayesian and the likelihood-based methods, interestingly corroborated an ancient founder origin for this variant, similar to what has been suggested for the *CHEK2* variant 1100delC [43,44]. Furthermore, the age estimates obtained by DMLE+2.3 for the distinct populations corroborate the likely origin of the variant in the Western/Central European region suggested by the haplotype phylogeographic distribution, approximately between 2015 and 2500 years ago. According to the results, it appears that the founder Western/Central population carrying distinct haplotypes, subsequently spread to the Iberian Peninsula, UK, and Scandinavia regions. The variant age estimates obtained for these populations are consistent with the European past population history, characterised by extensive movements in the first millennium, the so-called Migration Period or the Barbarian Invasions, which originated from the Central Europe region [45]. The haplotype analysis and age estimates results obtained for the American and Australian carriers are also in line of a European origin, most likely from British colonisers, who carried the European variant as they initially settled in those regions (Figure 3) [46,47]. Nevertheless, since these populations also shared the haplotype with other European populations that expanded to those continents, though to a lesser extent, we cannot rule out other possible origins.

The estimates of the variant age may oscillate, since it depends heavily on the population growth rates used, which historical evidence has shown to vary greatly over time. Therefore, in the present work, we tried to account for this caveat, by employing two different population growth rate estimates. However, caution is still needed when interpreting the age estimates, since the method relies on strong assumptions that cannot be entirely verified [48]. On the other hand, it is important to take into consideration that estimates based on historical population data may also contain errors that are difficult to account. Nevertheless, the age estimates obtained in the present study were consistent using different statistical approaches and are in line with the demographic history of the populations.

Taking into consideration that *CHEK2* variants have been previously associated with other types of cancer, such as breast cancer [22], we performed an additional microsatellite haplotype analysis of all carriers available at IPO-Porto. The microsatellite analysis further corroborated the founder nature of the *CHEK2* variant c.349A>G, by revealing the existence of a core haplotype shared among all 14 families carrying the variant, which is highly suggestive of a single mutational event rather than multiple independent events trough time. Furthermore, the fact that the variant was found in families with history of prostate, breast, gastric, and lung cancer supports *CHEK2* as a multiorgan cancer susceptibility gene, as previously suggested [49]. In fact, multiorgan susceptibly is characteristic of other genes in the DNA damage-signalling pathway, as has been observed for *BRCA1*, *BRCA2*, *PALB2*, and *ATM* genes [22,30,33,50,51,52,53].

## 4. Materials and Methods 

### 4.1. Portuguese Early-Onset/Familial PrCa Sample Collection

To clarify the possible association of the *CHEK2* variant c.349A>G with risk of early-onset/familial PrCa, we extended the genetic screening performed in a previous study of 121 cases [26] to the complete series of 462 cases with early-onset and/or familial PrCa [27]. As control data, we used the frequency obtained for 710 controls, previously described [26].

### 4.2. Genotyping of the CHEK2 Variant c.349A>G

Genotyping of the *CHEK2* variant c.349A>G was performed, in the 341 cases not previously screened, using the KASP technology genotyping (KBioscience, Herts, UK) with the KASP assay primers previously reported [26].

### 4.3. Statistical Analysis

To evaluate the cancer-associated risk of the *CHEK2* variant c.349A>G between cases and heathy controls in the Portuguese series of early-onset/familial PrCa and in the samples from the PRACTICAL Consortium, we estimated the odds ratios (ORs) and 95% confidence intervals (CIs) between carriers and non-carriers for the different studies. All analyses were carried out using R.

### 4.4. Practical Sample Collection 

For the high-density SNP haplotype analysis, we assembled genotype data obtained with the Infinium OncoArray-500K BeadChip (Illumina) for 93,746 participants from 54 studies, as part of the PRACTICAL consortium [4].

As this variant has only been reported in populations of European ancestral origin, we restricted the dataset to 55,162 PrCa cases and 36,147 controls of European ancestry from 53 studies. From the Portuguese early-onset/familial PrCa sample collection, 354 PrCa cases and 180 controls were included in the PRACTICAL final dataset. The detailed sample collection is described in Table S4. All studies were approved by the respective institutional review boards (38.010: Inherited predisposition to prostate cancer), and informed consent was obtained for all participants.

### 4.5. OncoArray Genotyping and Quality Control 

The OncoArray BeadChip includes a genome-wide backbone of 230,000 SNPs tagging most common genetic variants, and a customised panel of 250,000 SNPs developed from previous GWAS and fine-mapping studies of multiple cancer types, including PrCa [54]. The quality control of the high-density SNP data from chromosome 22 was performed as previously described [4]. Briefly, the procedure involved excluding SNPs with genotyping call rates <95% and failing Hardy–Weinberg equilibrium, as well as checking for duplicates/first-degree relatives and population ancestry using PLINK software [55]. We obtained a final dataset of 91,309 individuals and 8674 SNPs (Table S4), from here on termed as the PRACTICAL dataset.

### 4.6. Identity-By-Descent Analysis and Phylogeographic Haplotype Reconstruction

We performed identity-by-descent (IBD) and haplotype analysis for all carriers of the *CHEK2* variant c.349A>G in the PRACTICAL dataset. To obtain the population-matched control dataset for the downstream analyses, we corrected for population structure by pruning the PRACTICAL dataset to remove SNPs with excessive background linkage disequilibrium (pairwise genotypic correlation r^2^ > 0.4) within a 50-SNP sliding window in 10 SNP steps, and applied principal components analysis (PCA), with PLINK 1.9 and R software, to identify and exclude outliers (Figure S8). Then, we randomly reduced the control data from the variant-carrying populations to obtain a final dataset with 100 control individuals per population, except for the Netherlands (with only 65 controls).

High-density SNP data from chromosome 22 belonging to 1135 individuals (the 70 carriers and 1065 non-carriers of the variant) were phased using BEAGLE 4.1 [56]. The existence of shared haplotypes between carriers was assessed by IBD analysis using the Refined IBD algorithm [57]. The ibdtrim parameter was set to 25. The length of the shared haplotype was calculated by the distance between the two last shared markers flanking the *CHEK2* variant c.349A>G.

The phylogeographic patterns of variant-carrying haplotypes was determined by network reconstruction based on the median joining algorithm [58] using PopART v1.7 [59].

### 4.7. Age Estimation of the CHEK2 Variant c.349A>G

The SNPs flanking the margins of the different haplotypes identified by the IBD analysis, where recombination events were likely to have occurred, were selected for the estimation of the variant age using two statistical methods, the DMLE+2.3 [60] and ESTIAGE [61] software. The first method was used to estimate the age of the variant in the different populations separately, as well on the combined data of all populations to obtain an overall age estimate, whereas the second computational approach was only used to estimate the overall combined age of the variant, due to limitations of sample size per population group (as low as *n* = 4).

DMLE+2.3 uses a Bayesian method to compare differences in linkage disequilibrium between the variant and flanking markers in variant carriers and non-carriers. The software employs a Markov chain Monte Carlo (MCMC) method to generate the marginal posterior probability density of the variant age based on the observed haplotypes in variant-carrying or normal chromosomes; map distances between markers and variant site; population growth rates and an estimated proportion of the variant-carrying chromosomes sampled.

The population growth rates were estimated as described before, using the formula: $r\left(gen\right)=\frac{\ln\left(\frac{Pp}{P0}\right)}{g}$, where *r_(gen)_* represents the population growth rate per generation, *Pp* is the estimated present population size, *P*_0_ is the estimated size of the population at reference time, and *g* is the number of generations between these two time points (assuming 25 years per generation) [48,62]. Historical and current population size estimations were retrieved for all populations from Official Governmental demographic information (Table S5). In addition, since the formula mentioned above assumes a constant exponential population growth rate, which may not represent the history of the population, two rate estimates were employed to account for possible fluctuations. The overall rate (*r*_(*gen*)_1) was calculated using the oldest and the most recent population size estimates for each population, and the second, older rate (*r*_(*gen*)_2), was estimated using only the population sizes of each population until the beginning of last century.

The proportion of variant-carrying chromosomes sampled was estimated according to the frequency of the variant in each country (estimated based on the PRACTICAL dataset) and the number of existing males as of 2017 (Table S5).

The ESTIAGE implements a likelihood-based method to estimate the age of the most recent common ancestor (MRCA). We used allele frequencies obtained from control individuals and both stepwise and equal variant models with a variant rate of ≈2 × 10^−8^ at each marker [63].

The genetic distances (cM) used in both software were obtained from the 1000 Genomes Phase 3 data [64], and positions absent from this map were interpolated.

### 4.8. Microsatellite Analysis

To extend the haplotype analysis to the carriers of the recurrent *CHEK2* variant c.349A>G that were not genotyped using the OncoArray DNA chip, we used five polymorphic microsatellite markers flanking the gene, namely D22S310, D22S689, D22S275, D22S1150, and D22S280. A total of 14 probands carrying the *CHEK2* variant c.349A>G, which included an additional early-onset PrCa case not included in the initial PrCa series, and 18 family members were genotyped. Primer sequences (except for the D22S689 marker) were derived from the UCSC Genome Browser database (genome build 37) [65]. Primers for the D22S689 marker were designed using the online Primer-BLAST tool [66]. All markers were assayed by PCR using fluorescently end-labelled primers and PCR products were run on a 3500 Genetic Analyzer together with the fluorescence labelled DNA fragment size standard 600-LIZ (Thermo Fisher Scientific, Waltham, MA, USA). Haplotype construction was performed manually, based on the genotypes obtained from probands and family members.

## 5. Conclusions

Our results provide evidence that the c.349A>G variant in the *CHEK2* tumour-suppressor gene is significantly associated with increased risk of PrCa. Moreover, haplotype analysis using both high-density SNP and microsatellite data, as well as variant age estimates, strongly support a founder origin for this variant instead of multiple independent occurrences. The identification of founder variants, such as the one here reported, may contribute for the development of more cost-efficient screening strategies and counselling of high-risk families.

## Figures and Tables

**Table 1:** Microsatellite marker haplotypes of the 14 families carrying the *CHEK2* variant c.349A>G.

Microsatellite Markers						
Family	D22S310	D22S689	CHEK2	D22S275 (Intragenic)	D22S1150	D22S280
1 *	181	**294**	**_**	**159**	216	211
2 *	183	**294**	**_**	**159**	220	211
3 *	181	**294**	**_**	**159**	216	205/211
4 *	179	**294**	**_**	**159**	216/220	205
5 *	183/187	290/**294**	_	**159**/163	218/220	205/211
6	185/189	**294**	_	**159**/161	216/220	205
7	187	**294**	_	**159**	216	211
8	185	**294**	_	**159**	216	211
9	177/185	**294**/298	_	**159**/161	220	209
10	179/187	**294**	_	**159**/167	218/220	205/209
11	177/189	**294**	_	**159**	216	205
12	185	**294**	_	**159**	216	205/209
13	185	294	_	**159**	220	209
14	177/181	**294**/302	_	**159**	216/226	205/213

* Included in the high-density SNP haplotype analysis performed with the PRACTICAL samples. The shared core haplotype associated with the variant is represented in bold.

## Supplementary Materials

The following are available online at https://www.mdpi.com/2072-6694/12/11/3254/s1, Figure S1: Age estimation of the *CHEK2* variant c.349A>G in the Western/Central Europe populations (France, Belgium, Germany, and Netherlands) using the DMLE 2.3 software, Figure S2: Age estimation of the *CHEK2* variant c.349A>G in the Spanish carriers using the DMLE 2.3 software, Figure S3: Age estimation of the *CHEK2* variant c.349A>G in the Portuguese carriers using the DMLE 2.3 software, Figure S4: Age estimation of the *CHEK2* variant c.349A>G in the British carriers using the DMLE 2.3 software, Figure S5: Age estimation of the *CHEK2* variant c.349A>G in the Scandinavian carriers (Denmark and Sweden) using the DMLE 2.3 software, Figure S6: Age estimation of the *CHEK2* variant c.349A>G in the Western/Central Europe populations (France, Belgium, Germany, and Netherlands) using the DMLE 2.3 software, Figure S7: Age estimation of the *CHEK2* variant c.349A>G in U.S. carriers using the DMLE 2.3 software, Figure S8: PCA plots of all *CHEK2* variant c.349A>G carriers along with control individuals from the 11 variant-carrying populations (Australia, Belgium, Denmark, France, Germany, Netherlands, Portugal, Spain, Sweden, UK, and USA) and China, the outlier population for the PCA analysis, Table S1: Characterisation of the populations carrying the *CHEK2* variant c.349A>G included in the PRACTICAL consortium, Table S2: Overall age estimates of the *CHEK2* variant c.349A>G, Table S3: Age estimates of the *CHEK2* variant c.349A>G in the different populations, Table S4: Characterisation of the studies and participants from PRACTICAL consortium, Table S5: Population information for population growth rates estimation. Supplementary Information: supplementary authors, additional funding and acknowledgments.
