# Supplementary material for: The CHEK2 Variant C.349A>G Is Associated with Prostate Cancer Risk and Carriers Share a Common Ancestor
Source: Cancers (Basel). 2020 Nov 4;12(11):3254. doi: 10.3390/cancers12113254 (PMC7694218; doi:10.3390/cancers12113254)
Supplement: Supplementary file 1 [file cancers-12-03254-s001.zip › cancers-950280-XML suppl/cancers-950280-XML suppl tables and figures.docx]

Supplemental Materials

The *CHEK2* Variant C.349A>G Is Associated with Prostate Cancer Risk and Carriers Share a Common Ancestor

Andreia Brandão, Paula Paulo, Sofia Maia, Manuela Pinheiro, Ana Peixoto, Marta Cardoso, Maria P. Silva, Catarina Santos, Rosalind A. Eeles, Zsofia Kote-Jarai, Kenneth Muir, UKGPCS collaborators, Johanna Schleutker, Ying Wang, Nora Pashayan, Jyotsna Batra, APCB BioResource, Henrik Grönberg, David E. Neal, Børge G. Nordestgaard, Catherine M. Tangen, Melissa C. Southey, Alicja Wolk, Demetrius Albanes, Christopher A. Haiman, Ruth C. Travis, Janet L. Stanford, Lorelei A. Mucci, Catharine M. L. West, Sune F. Nielsen, Adam S. Kibel, Olivier Cussenot, Sonja I. Berndt, Stella Koutros, Karina Dalsgaard Sørensen, Cezary Cybulski, Eli Marie Grindedal, Jong Y. Park, Sue A. Ingles, Christiane Maier, Robert J. Hamilton, Barry S. Rosenstein, Ana Vega, The IMPACT Study Steering Committee and Collaborators, Manolis Kogevinas, Fredrik Wiklund, Kathryn L. Penney, Hermann Brenner, Esther M. John, Radka Kaneva, Christopher J. Logothetis, Susan L. Neuhausen, Kim De Ruyck, Azad Razack, Lisa F. Newcomb, Canary PASS Investigators, Davor Lessel, Nawaid Usmani, Frank Claessens, Manuela Gago-Dominguez, Paul A. Townsend, Monique J. Roobol, The Profile Study Steering Committee, The PRACTICAL Consortium and Manuel R. Teixeira

**Table S1.** Characterization of the populations carrying the *CHEK2* variant c.349A>G included in the PRACTICAL consortium.

| Study Acronyms | Country | Cases | Controls | Total | Total Sampled |
| --- | --- | --- | --- | --- | --- |
| CPCS1 | Denmark | 0 | 1 | 1 | 3098 |
| CPS-II; HPFS; PHS; SFPCS; SWOG-SELECT; WUGS; Gene-PARE (USA) | USA | 10 | 3 | 13 | 30853 |
| QLD | Australia | 4 | 0 | 4 | 5616 |
| ProMPT; RAPPER SEARCH; UKGPCS | UK | 18 | 4 | 22 | 28639 |
| ULM | Germany | 1 | 0 | 1 | 1275 |
| COSM; PROCA; STHM2 | Sweden | 6 | 4 | 10 | 8846 |
| SNP_Prostate_Ghent | Belgium | 0 | 1 | 1 | 720 |
| CeRePP | France | 2 | 1 | 3 | 1570 |
| PROGReSS | Spain | 7 | 2 | 9 | 2150 |
| ERSPC | Netherlands | 0 | 1 | 1 | 137 |
| IPO-Porto | Portugal | 4 | 1 | 5 | 554 |

**Table S2.** Overall age estimates of the *CHEK2* variant c.349A>G**.**

| Method | Generations  (95% CI) | Years  (95% CI) | Generations  (95% CI) | Years  (95% CI) | Generations  (95% CI) | Years  (95% CI) |
| --- | --- | --- | --- | --- | --- | --- |
| DMLE 2.3 | **r_(gen)_ 1** | | **r_(gen)_ 2** | | **Average age estimates** | |
|  | 113  (97–145) | 2825  (2425–3625) | 92  (78–118) | 2300  (1950–2950) | 103  (88–132) | 2563  (2188–3288) |
| ESTIAGE | **Equal model** | | **Stepwise model** | | 124  (105–147) | 3100  (2625–3675) |
|  | 125  (106–148) | 3125  (2650–3700) | 123  (104–146) | 3075  (2600–3650) |  |  |

**Table S3.** Age estimates of the *CHEK2* variant c.349A>G in the different populations.

| Populations | r_(gen)_ 1 | | r_(gen)_ 2 | | Average age estimates | |
| --- | --- | --- | --- | --- | --- | --- |
|  | **Generations (95% CI)** | **Years**  **(95% CI)** | **Generations (95% CI)** | **Years (95% CI)** | **Generations (95% CI)** | **Years (95% CI)** |
| Central/Western Europe (France; Belgium; Germany; Netherlands) | 100  (81–132) | 2500  (2025–3300) | 81  (65–104) | 2015  (1625–2600) | 91  (73–118) | 2258  (1825–2950) |
| Spain | 61  (49–78) | 1525  (1225–1950) | 73  (61–96) | 1825  (1525–2400) | 67  (55–87) | 1675  (1375–2175) |
| Portugal | 61  (48–83) | 1525  (1200–2075) | 53  (42–72) | 1325  (1050–1800) | 57  (45–78) | 1425  (1125–1938) |
| Scandinavia (Denmark; Sweden) | 49  (38–70) | 1225  (950–1750) | 42  (31–59) | 1050  (775–1475) | 46  (35–65) | 1138  (863–1613) |
| UK | 54  (43–71) | 1350  (1075–1775) | 33  (27–44) | 825  (675–1100) | 44  (35–58) | 1088  (875–14378) |
| Australia | 27  (21–38) | 675  (525–950) | 18  (13–24) | 450  (325–600) | 23  (17–31) | 563  (425–775) |
| USA | 23  (20–29) | 575  (500–725) | 16  (14–21) | 400  (350–525) | 20  (17–25) | 488  (425–625) |

**Table S4.** Characterization of the studies and participants from PRACTICAL consortium.

| Institution | Study Acronym | Country | Cases | Control | Total |
| --- | --- | --- | --- | --- | --- |
| Aarhus University Hospital | Aarhus | Denmark | 1077 | 545 | 1622 |
| National Cancer Institute, National Institutes of Health | AHS | USA | 491 | 1159 | 1650 |
| National Cancer Institute, National Institutes of Health | ATBC | USA | 1281 | 1913 | 3194 |
| Fred Hutchinson Cancer Research Center | Canary PASS | USA | 364 | 0 | 364 |
| Alberta Health Services | CCI | Canada | 266 | 0 | 266 |
| Centre for Research on Prostatic Diseases (CeRePP) | CeRePP | France | 923 | 644 | 1567 |
| Beckman Research Institute of the City of Hope | COH | USA | 257 | 259 | 516 |
| Karolinska Institutet | COSM | Sweden | 2298 | 1117 | 3415 |
| Herlev University Hospital | CPCS1 | Denmark | 536 | 258 | 794 |
| Herlev University Hospital | CPCS2 | Denmark | 444 | 228 | 672 |
| American Cancer Society, Atlanta | CPS-II | USA | 4401 | 4063 | 8464 |
| University of Oxford | EPIC | Multi Center in EU | 635 | 693 | 1328 |
| Erasmus MC | ERSPC | The Netherlands | 71 | 65 | 136 |
| German Cancer Research Center | ESTHER | Germany | 324 | 315 | 639 |
| Fred Hutchinson Cancer Research Center | FHCRC | USA | 407 | 388 | 795 |
| Icahn School of Medicine at Mount Sinai (Radiogenomics) | Gene-PARE | USA and Japan | 242 | 0 | 242 |
| University Medical Center Hamburg-Eppendorf | Hamburg-Zagreb | Germany | 146 | 149 | 295 |
| Harvard School of Public Health | HPFS | USA | 1168 | 1044 | 2212 |
| The Institute of Cancer Research | IMPACT | UK | 49 | 867 | 916 |
| Instituto Português de Oncologia do Porto Francisco Gentil | IPO-Porto | Portugal | 374 | 180 | 554 |
| University of Leuven | KULEUVEN | Belgium | 166 | 103 | 269 |
| University of Southern California | LAAPC | USA | 440 | 280 | 720 |
| University of Malaya | Malaysia | Malaysia | 1 | 0 | 1 |
| Cancer Council Victoria | MCCS | Australia | 715 | 315 | 1030 |
| Barcelona Institute for Global Health (ISGlobal) | MCC-Spain | Spain | 520 | 397 | 917 |
| The University of Texas, MD Anderson Cancer Center | MDACC_AS | USA | 501 | 0 | 501 |
| University of Southern California & University of Hawai | MEC | USA | 598 | 642 | 1240 |
| H. Lee Moffitt Cancer Center and Research Institute | MOFFITT | USA | 403 | 203 | 606 |
| Oslo University Hospital | Oslo | Norway | 1443 | 0 | 1443 |
| Medical University of Sofia | PCMUS | Bulgaria | 192 | 89 | 281 |
| Brigham and Women’s Hospital | PHS | USA | 622 | 257 | 879 |
| National Cancer Institute, National Institutes of Health | PLCO | USA | 678 | 980 | 1658 |
| Pomeranian Medical University, Szczecin | Poland | Poland | 484 | 317 | 801 |
| Galician Foundation of Genomic Medicine, Santiago de Compostela | PRAGGA | Spain | 129 | 100 | 229 |
| Karolinska Institutet | PROCAP | Sweden | 659 | 236 | 895 |
| The Institute of Cancer Research | PROFILE | UK | 13 | 21 | 34 |
| Fundacion Publica Gallega Medicina Xenomica, Santiago de Compostela | PROGReSS | Spain | 673 | 322 | 995 |
| University of Cambridge, University of Bristol & University of Oxford | ProMPT | UK | 839 | 12 | 851 |
| University of Cambridge, University of Bristol & University of Oxford | ProtecT | UK | 4 | 1409 | 1413 |
| Queensland University of Technology | QLD | Australia | 3282 | 1241 | 4523 |
| The University of Manchester | RAPPER | UK | 2138 |  | 2138 |
| University of Cambridge | SEARCH | UK | 2511 | 1442 | 3953 |
| Cancer Prevention Institute of California and Stanford University School of Medicine | SFPCS | USA | 279 | 205 | 484 |
| The Ghent University | SNP_Prostate_Ghent | Belgium | 316 | 135 | 451 |
| The University of Surrey | SPAG | UK | 39 | 171 | 210 |
| Karolinska Institutet | STHM2 | Sweden | 3019 | 1481 | 4500 |
| SWOG Statistical Center, Fred Hutchinson Cancer Research Center | SWOG-PCPT | USA | 1072 | 1084 | 2156 |
| SWOG Statistical Center, Fred Hutchinson Cancer Research Center | SWOG-SELECT | USA | 1479 | 2070 | 3549 |
| University of Tampere | TAMPERE | Finland | 2421 | 1183 | 3604 |
| Princess Margaret Hospital, Toronto | TORONTO | Canada | 668 | 455 | 1123 |
| The Institute of Cancer Research | UKGPCS | UK | 11971 | 6932 | 18903 |
| University Hospital of Ulm | ULM | Germany | 457 | 178 | 635 |
| Washington University School of Medicine | WUGS | USA | 676 | 0 | 676 |

**Table S5.** Population information for population growth rates estimation.

| Countries | r_(gen)_ 1 | r_(gen)_ 2 | ƒ | Government Information Reference | Additonal Resources References |
| --- | --- | --- | --- | --- | --- |
| Portugal | 0.15 | 0.17 | 0.00012 | [www.ine.pt](http://www.ine.pt/) | [www.ec.europa.eu/eurostat](http://www.ec.europa.eu/eurostat) |
| Spain | 0.17 | 0.14 | 0,00009 | [www.ine.es](http://www.ine.es/) |  |
| Western/Central Europe (France, Belgium, Germany and Netherlands) | 0.10 | 0.13 | 0.00004 | [www.insee.fr](http://www.insee.fr/) | [www.visionofbritain.org.uk](http://www.visionofbritain.org.uk/) |
|  |  |  |  | [www.statbel.fgov.be](http://www.statbel.fgov.be/) | [www.cia.gov/library/publications/resources/the-world-factbook](http://www.cia.gov/library/publications/resources/the-world-factbook) |
|  |  |  |  | [www.destatis.de](http://www.destatis.de/) | [www.populstat.info](http://www.populstat.info/) |
|  |  |  |  | [www.cbs.nl](http://www.cbs.nl/) | [www.population.un.org](http://www.population.un.org/) |
| Scandinavia  (Denmark and Sweden) | 0.15 | 0.18 | 0.00152 | [www.dst.dk](http://www.dst.dk/) |  |
|  |  |  |  | [www.scb.se](http://www.scb.se/) |  |
| United Kingdom | 0.17 | 0.29 | 0.00089 | [www.ons.gov.uk](http://www.ons.gov.uk/) |  |
| United States of America | 0.50 | 0.67 | 0,00025 | [www.census.gov](http://www.census.gov/) |  |
| Australia | 0.40 | 0.48 | 0.00049 | [www.abs.gov.au](http://www.abs.gov.au/) |  |
| All Populations | 0.10 | 0.13 | 0.00028 |  |  |

**r_(gen)_ 1 –** Population growth rates estimated based on the oldest and the most recent census.

**r_(gen)_ 2 –** Population growth rates estimated based on census data until 1900.

***ƒ –*** Estimated variant-carrying sampled proportion.

**Figure S1.** Age estimation of the *CHEK2* variant c.349A>G in the Western/Central Europe populations (France, Belgium, Germany and Netherlands) using the DMLE 2.3 software. Distribution of the posterior probability for the age estimation, assuming 0.00004 as the proportion of variant-bearing chromosomes and two population growth rates 0.10 and 0.13. The maximum likelihood estimated the age of the mutation between 81 (95% CI: 65–104) and 100 (95% CI: 81–132) generations ago. Considering a generation length of 25 years, this finding translates in an age between approximately 2015 and 2500 years.

**Figure S2.** Age estimation of the *CHEK2* variant c.349A>G in the Spanish carriers using the DMLE 2.3 software. Distribution of the posterior probability for the age estimation, assuming 0.00009 as the proportion of variant-bearing chromosomes and two population growth rates 0.14 and 0.17. The maximum likelihood estimated the age of the mutation between 61 (95% CI: 49–78) and 73 (95% CI: 61–96) generations ago. Considering a generation length of 25 years, this finding translates in an age between approximately 1525 and 1825 years.

**Figure S3.** Age estimation of the *CHEK2* variant c.349A>G in the Portuguese carriers using the DMLE 2.3 software. Distribution of the posterior probability for the age estimation, assuming 0.00012 as the proportion of variant-bearing chromosomes and two population growth rates 0.15 and 0.17. The maximum likelihood estimated the age of the mutation between 53 (95% CI: 42–72) and 61 (95% CI: 48–83) generations ago. Considering a generation length of 25 years, this finding translates in an age between approximately 1325 and 1525 years.

**Figure S4.** Age estimation of the *CHEK2* variant c.349A>G in the British carriers using the DMLE 2.3 software. Distribution of the posterior probability for the age estimation, assuming 0.00089 as the proportion of variant-bearing chromosomes and two population growth rates 0.17 and 0.29. The maximum likelihood estimated the age of the mutation between 33 (95% CI: 27–44) and 54 (95% CI: 43–71) generations ago. Considering a generation length of 25 years, this finding translates in an age between approximately 825 and 1350 years.

**Figure S5.** Age estimation of the *CHEK2* variant c.349A>G in the Scandinavian carriers (Denmark and Sweden) using the DMLE 2.3 software. Distribution of the posterior probability for the age estimation, assuming 0.00152 as the proportion of variant-bearing chromosomes and two population growth rates 0.15 and 0.18. The maximum likelihood estimated the age of the mutation between 42 (95% CI: 31–59) and 49 (95% CI: 38–70) generations ago. Considering a generation length of 25 years, this finding translates in an age between approximately 1050 and 1225 years.

**Figure S6.** Age estimation of the *CHEK2* variant c.349A>G in the Western/Central Europe populations (France, Belgium, Germany and Netherlands) using the DMLE 2.3 software. Distribution of the posterior probability for the age estimation, assuming 0.00049 as the proportion of variant-bearing chromosomes and two population growth rates 0.40 and 0.48. The maximum likelihood estimated the age of the mutation between 18 (95% CI: 13–24) and 27 (95% CI: 21–38) generations ago. Considering a generation length of 25 years, this finding translates in an age between approximately 400 and 675 years.

**Figure S7.** Age estimation of the *CHEK2* variant c.349A>G in U.S. carriers using the DMLE 2.3 software. Distribution of the posterior probability for the age estimation, assuming 0.00025 as the proportion of variant-bearing chromosomes and two population growth rates 0.50 and 0.67. The maximum likelihood estimated the age of the mutation between 16 (95% CI: 14–21) and 23 (95% CI: 20–29) generations ago. Considering a generation length of 25 years, this finding translates in an age between approximately 400 and 575 years.


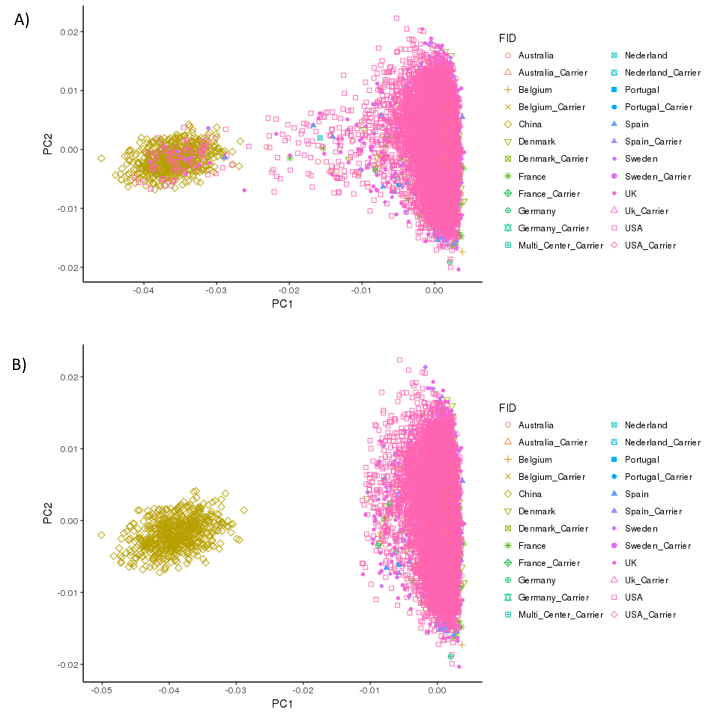


**Figure S8.** PCA plots of all *CHEK2* variant c.349A>G carriers along with control individuals from the 11 variant-carrying populations (Australia, Belgium, Denmark, France, Germany, Netherlands, Portugal, Spain, Sweden, UK and USA) and China, the outlier population for the PCA analysis. A) PCA analysis before correcting for populations stratification. B) PCA analysis after correcting for population stratification. Principal component 1 is displayed on the *x* axis, and principal component 2 is displayed on the *y* axis.
