# Supplementary material for: The CHEK2 Variant C.349A>G Is Associated with Prostate Cancer Risk and Carriers Share a Common Ancestor
Source: Cancers (Basel). 2020 Nov 4;12(11):3254. doi: 10.3390/cancers12113254 (PMC7694218; doi:10.3390/cancers12113254)
Supplement: Supplementary file 1 [file cancers-12-03254-s001.zip › cancers-950280-XML suppl/cancers-950280-XML-Suppl authors.docx]

**Supplementary Information**

**Brandão et al.**

**The *CHEK2* variant c.349A>G is associated with prostate cancer risk and carriers share a common ancestor**

**1. Supplementary authors from The PRACTICAL (http://practical.icr.ac.uk/) Consortium:**

Artitaya Lophatananon^1^, Teuvo L. J. Tammela^2,3^, Csilla Sipeky^4^, Anssi Auvinen^5^, Brian D. Carter^6^, Alison M. Dunning^7^, Maya Ghoussaini^8^, Suzanne Chambers^9,10^, Lisa Horvath^11,12^, Leire Moya^13,14^, Gail P. Risbridger^15,16^, Wayne Tilley^17^, Judith A. Clements^13,14^, Markus Aly^18,19,20^, Tobias Nordström^18,21^, Jenny L. Donovan^22^, Freddie C. Hamdy^23,24^, Richard M. Martin^22,25,26^, Stig E. Bojesen^27,28^, Peter Iversen^29^, Martin Andreas Røder^29^, Phyllis J. Goodman^30^, Ian M. Thompson Jr.^31^ , Graham G. Giles^32,33,34^, Robert J. MacInnis^32,33^, Roger L. Milne^32,33,34^, Niclas Håkansson^35^, Stephanie Weinstein^36^, Fredrick R. Schumacher^37,38^, Loic Le Marchand^39^, Xin Sheng^40^, Tim J. Key^41^, Elaine A. Ostrander^42^, Edward Giovannucci^43^, Neil Burnet^44^, Gill Barnett^45^, Bettina F. Drake^46^, Géraldine Cancel-Tassin^47,48^, Stephen Chanock^36^, Gerald L. Andriole^46^, Robert N. Hoover^36^, Mitchell J. Machiela^36^, Laura E. Beane Freeman^36^, Michael Borre^49,50^, Dominika Wokolorczyk^51^, Jan Lubinski^51^, Lovise Maehle^52^, Hui-Yi Lin^53^, Mariana C Stern^54^, Manuel Luedeke^55^, Thomas Schnoeller^56^, Neil E. Fleshner^57^, Antonio Finelli^58^, Sarah L. Kerns^59^, Harry Ostrer^60^, Antonio Gómez-Caamaño^61^, Laura Fachal^62,63,64,65^, Javier Llorca^66,67^, Gemma Castaño-Vinyals^68,69,70,71^, Antonio Alcaraz^72^, Peter Kraft^73^, Sara Lindström^74^, Meir Stampfer^75^, Xin Gao^76^, Bernd Holleczek^77^, Ben Schöttker^76^, Chavdar Slavov^78^, Vanio Mitev^79^, Jeri Kim^80^, Yuan Chun Ding^81^, Linda Steele^81^, Gert De Meerleer^82^, Piet Ost^83^, Jasmine Lim^84^, Soo-Hwang Teo^85^, Daniel W. Lin^86,87^,Marija Gamulin^88^, Tomislav Kulis^89^, Matthew Parliament^90,91^, Aswin Abraham^90^, Steven Joniau^92^, Thomas Van den Broeck^93,92^, Jose Esteban Castelao^94^, Maria Elena Martinez^95^, Ron H.N. van Schaik^96^, Guido Jenster^97^

^1^Division of Population Health, Health Services Research and Primary Care, University of Manchester, Oxford Road, Manchester, M13 9PL, UK
^2^Department of Urology, Tampere University Hospital, FI-33521 Tampere, Finland
^3^Faculty of Medicine and Health Technology, Tampere University, FI-33100 Tampere, Finland
^4^Institute of Biomedicine, University of Turku, Finland
^5^Unit of Health Sciences, Faculty of Social Sciences, Tampere University, Tampere, Finland
^6^Department of Population Science, American Cancer Society, 250 Williams Street, Atlanta, GA 30303, USA
^7^Centre for Cancer Genetic Epidemiology, Department of Oncology, University of Cambridge, Strangeways Laboratory, Worts Causeway, Cambridge, CB1 8RN, UK
^8^Open Targets, Wellcome Sanger Institute, Hinxton, Saffron Walden, CB10 1SA, UK
^9^University of Technology, Sydney
^10^Cancer Council Queensland, Fortitude Valley, QLD 4006, Australia
^11^Chris O'Brien Lifehouse (COBLH), Camperdown, Sydney, NSW 2010, Australia
^12^Garvan Institute of Medical Research, Sydney NSW 2010, Australia
^13^Australian Prostate Cancer Research Centre-Qld, Institute of Health and Biomedical Innovation and School of Biomedical Sciences, Queensland University of Technology, Brisbane QLD 4059, Australia
^14^Translational Research Institute, Brisbane, Queensland 4102, Australia
^15^Department of Anatomy and Developmental Biology, Biomedicine Discovery Institute, Monash University, Melbourne,Victoria 3800, Australia
^16^Prostate Cancer Translational Research Program, Cancer Research Division, Peter MacCallum Cancer Centre, Melbourne, VIC 3000, Australia
^17^Dame Roma Mitchell Cancer Research Laboratories, University of Adelaide, Adelaide, South Australia, Australia
^18^Department of Medical Epidemiology and Biostatistics, Karolinska Institute, SE-171 77 Stockholm, Sweden
^19^Department of Molecular Medicine and Surgery, Karolinska Institutet
^20^Department of Urology, Karolinska University Hospital, Solna, 171 76 Stockholm
^21^Department of Clinical Sciences at Danderyds Hospital, Karolinska Institutet, Stockholm, Sweden
^22^Population Health Sciences, Bristol Medical School, University of Bristol, BS8 2PS, UK
^23^Nuffield Department of Surgical Sciences, University of Oxford, Oxford, OX1 2JD, UK
^24^Faculty of Medical Science, University of Oxford, John Radcliffe Hospital, Oxford, UK
^25^National Institute for Health Research (NIHR) Biomedical Research Centre, University of Bristol, Bristol, BS8 1TH, UK
^26^Medical Research Council (MRC) Integrative Epidemiology Unit, University of Bristol, Bristol, BS8 2BN, UK.
^27^Faculty of Health and Medical Sciences, University of Copenhagen, 2200 Copenhagen, Denmark
^28^Department of Clinical Biochemistry, Herlev and Gentofte Hospital, Copenhagen University Hospital, Herlev, 2200 Copenhagen, Denmark
^29^Copenhagen Prostate Cancer Center, Department of Urology, Rigshospitalet, Copenhagen University Hospital, DK-2730 Herlev, Copenhagen, Denmark
^30^SWOG Statistical Center, Fred Hutchinson Cancer Research Center, Seattle, WA, USA
^31^CHRISTUS Santa Rosa Hospital – Medical Center, San Antonio, TX, USA

^32^Cancer Epidemiology Division, Cancer Council Victoria, 615 St Kilda Road, Melbourne, VIC 3004, Australia

^33^Centre for Epidemiology and Biostatistics, Melbourne School of Population and Global Health, The University of Melbourne, Grattan Street, Parkville, VIC 3010, Australia

^34^Precision Medicine, School of Clinical Sciences at Monash Health, Monash University, Clayton, Victoria 3168, Australia

^35^Unit of Cardiovascular and Nutritional Epidemiology, Institute of Environmental Medicine, Karolinska Institutet, SE-171 77 Stockholm, Sweden
^36^Division of Cancer Epidemiology and Genetics, National Cancer Institute, NIH, Bethesda, Maryland, 20892, USA
^37^Department of Population and Quantitative Health Sciences, Case Western Reserve University, Cleveland, OH 44106-7219, USA
^38^Seidman Cancer Center, University Hospitals, Cleveland, OH 44106, USA.
^39^Epidemiology Program, University of Hawaii Cancer Center, Honolulu, HI 96813, USA
^40^Center for Genetic Epidemiology, Department of Preventive Medicine, Keck School of Medicine, University of Southern California/Norris Comprehensive Cancer Center, Los Angeles, CA 90015, USA
^41^Cancer Epidemiology Unit, Nuffield Department of Population Health, University of Oxford, Oxford, OX3 7LF, UK
^42^National Human Genome Research Institute, National Institutes of Health, 50 South Drive, Rm. 5351, Bethesda, MD 20892, USA
^43^Department of Epidemiology, Harvard School of Public Health, Boston, MA 02115, USA
^44^Division of Cancer Sciences, University of Manchester, Manchester Cancer Research Centre, Manchester Academic Health Science Centre, and The Christie NHS Foundation Trust, Manchester, UK
^45^University of Cambridge, Department of Oncology, Oncology Centre, Cambridge University Hospitals NHS Foundation Trust, Hills Road, Cambridge, CB2 0QQ, UK
^46^Washington University School of Medicine, 660 S. Euclid Avenue, Campus Box 8242, St. Louis, MO 63110 , USA
^47^CeRePP, Tenon Hospital, F-75020 Paris, France.
^48^Sorbonne Universite, GRC n°5 , AP-HP, Tenon Hospital, 4 rue de la Chine, F-75020 Paris, France
^49^Department of Urology, Aarhus University Hospital, Palle Juul-Jensen Boulevard 99, 8200 Aarhus N, Denmark
^50^Department of Clinical Medicine, Aarhus University, DK-8200 Aarhus N
^51^International Hereditary Cancer Center, Department of Genetics and Pathology, Pomeranian Medical University, 70-115 Szczecin, Poland
^52^Department of Medical Genetics, Oslo University Hospital, 0424 Oslo, Norway
^53^School of Public Health, Louisiana State University Health Sciences Center, New Orleans, LA 70112, USA
^54^Department of Preventive Medicine, Keck School of Medicine, University of Southern California/Norris Comprehensive Cancer Center, Los Angeles, CA 90015, USA
^55^Humangenetik Tuebingen, Paul-Ehrlich-Str 23, D-72076 Tuebingen, Germany
^56^Department of Urology, University Hospital Ulm, Germany
^57^Dept. of Surgical Oncology, Princess Margaret Cancer Centre, Toronto ON M5G 2M9, Canada
^58^Division of Urology, Princess Margaret Cancer Centre, Toronto ON M5G 2M9, Canada
^59^Department of Radiation Oncology, University of Rochester Medical Center, Saunders Research Building, Rm 2.202, 265 Crittenden Boulevard, Rochester, NY 14620, USA
^60^Professor of Pathology and Pediatrics, Albert Einstein College of Medicine, 1300 Morris Park Avenue, Ullman 817, Bronx, NY 10461, USA
^61^Department of Radiation Oncology, Complexo Hospitalario Universitario de Santiago, SERGAS, Santiago de Compostela 15706, Spain
^62^Centre for Cancer Genetic Epidemiology, Department of Public Health and Primary Care, University of Cambridge, Strangeways Research Laboratory, Cambridge, CB2 0SR, UK.
^63^Fundación Pública Galega Medicina Xenómica, Santiago De Compostela, 15706, Spain.
^64^Instituto de Investigación Sanitaria de Santiago de Compostela, Santiago De Compostela, 15706, Spain.
^65^Centro de Investigación en Red de Enfermedades Raras (CIBERER), Spain
^66^University of Cantabria-IDIVAL, 39005 Santander, Spain
^67^CIBER Epidemiología y Salud Pública (CIBERESP), 28029 Madrid, Spain
^68^ISGlobal, Barcelona, Spain
^69^IMIM (Hospital del Mar Medical Research Institute), Barcelona, Spain
^70^Universitat Pompeu Fabra (UPF), Barcelona, Spain
^71^CIBER Epidemiología y Salud Pública (CIBERESP), Madrid, Spain
^72^Department and Laboratory of Urology. Hospital Clínic. Institut d’Investigacions Biomèdiques August Pi i Sunyer (IDIBAPS), Universitat de Barcelona. Spain. C/Villarroel 170; 08036 Barcelona, Spain
^73^Program in Genetic Epidemiology and Statistical Genetics, Department of Epidemiology, Harvard School of Public Health, Boston, MA, USA
^74^Department of Epidemiology, Health Sciences Building, University of Washington
^75^Channing Division of Network Medicine, Department of Medicine, Brigham and Women's Hospital/Harvard Medical School, Boston, MA 02184, USA
^76^Division of Clinical Epidemiology and Aging Research, German Cancer Research Center (DKFZ), D-69120, Heidelberg, Germany
^77^Saarland Cancer Registry, 66119 Saarbrücken, Germany
^78^Department of Urology and Alexandrovska University Hospital, Medical University of Sofia, 1431 Sofia, Bulgaria
^79^Molecular Medicine Center, Department of Medical Chemistry and Biochemistry, Medical University of Sofia, Sofia, 2 Zdrave Str., 1431 Sofia, Bulgaria
^80^The University of Texas M. D. Anderson Cancer Center, Department of Genitourinary Medical Oncology, 1515 Holcombe Blvd., Houston, TX 77030, USA
^81^Department of Population Sciences, Beckman Research Institute of the City of Hope, 1500 East Duarte Road, Duarte, CA 91010, 626-256-HOPE (4673)
^82^Ghent University, B-9000, Gent
^83^Ghent University Hospital, Department of Radiotherapy, De Pintelaan 185, B-9000, Gent, Belgium
^84^Department of Surgery, Faculty of Medicine, University of Malaya, 50603 Kuala Lumpur, Malaysia
^85^Cancer Research Malaysia (CRM), Outpatient Centre, Subang Jaya Medical Centre, Subang Jaya, Selangor, Malaysia
^86^Division of Public Health Sciences, Fred Hutchinson Cancer Research Center, Seattle, Washington, 98109-1024, USA
^87^Department of Urology, University of Washington, 1959 NE Pacific Street, Box 356510, Seattle, WA 98195, USA
^88^Division of Medical Oncology, Urogenital Unit, Department of Oncology, University Hospital Centre Zagreb, University of Zagreb, School of Medicine, 10 000 Zagreb, Croatia
^89^Department of Urology, University Hospital Center Zagreb, University of Zagreb School of Medicine , Zagreb, Croatia
^90^Department of Oncology, Cross Cancer Institute, University of Alberta, 11560 University Avenue, Edmonton, Alberta, Canada T6G 1Z2
^91^Division of Radiation Oncology, Cross Cancer Institute, 11560 University Avenue, Edmonton, Alberta, Canada T6G 1Z2
^92^Department of Urology, University Hospitals Leuven, Herestraat 49, Box 7003 41, BE-3000 Leuven, Belgium
^93^Molecular Endocrinology Laboratory, Department of Cellular and Molecular Medicine, KU Leuven, BE-3000, Belgium
^94^Genetic Oncology Unit, CHUVI Hospital, Complexo Hospitalario Universitario de Vigo, Instituto de Investigación Biomédica Galicia Sur (IISGS), 36204, Vigo (Pontevedra), Spain
^95^University of California San Diego, Moores Cancer Center, Department of Family Medicine and Public Health, University of California San Diego, La Jolla, CA 92093-0012, USA
^96^Department of Clinical Chemistry, Erasmus University Medical Center, 3015 CE Rotterdam, The Netherlands
^97^Department of Urology, Erasmus University Medical Center, 3015 CE Rotterdam, The Netherlands

**2. Additional funding and acknowledgments from studies in PRACTICAL:**

Information on The PRACTICAL Consortium can be found at <http://practical.icr.ac.uk/>

The PRACTICAL consortium

This work was supported by the Canadian Institutes of Health Research, European Commission's Seventh Framework Programme grant agreement n° 223175 (HEALTH-F2-2009-223175), Cancer Research UK Grants C5047/A7357, C1287/A10118, C1287/A16563, C5047/A3354, C5047/A10692, C16913/A6135, and The National Institute of Health (NIH) Cancer Post-Cancer GWAS initiative grant: No. 1 U19 CA 148537-01 (the GAME-ON initiative).

We would also like to thank the following for funding support: The Institute of Cancer Research and The Everyman Campaign, The Prostate Cancer Research Foundation, Prostate Research Campaign UK (now PCUK), The Orchid Cancer Appeal, Rosetrees Trust, The National Cancer Research Network UK, The National Cancer Research Institute (NCRI) UK. We are grateful for support of NIHR funding to the NIHR Biomedical Research Centre at The Institute of Cancer Research and The Royal Marsden NHS Foundation Trust.
The Prostate Cancer Program of Cancer Council Victoria also acknowledge grant support from The National Health and Medical Research Council, Australia (126402, 209057, 251533, , 396414, 450104, 504700, 504702, 504715, 623204, 940394, 614296,), VicHealth, Cancer Council Victoria, The Prostate Cancer Foundation of Australia, The Whitten Foundation, PricewaterhouseCoopers, and Tattersall’s. EAO, DMK, and EMK acknowledge the Intramural Program of the National Human Genome Research Institute for their support.

Genotyping of the OncoArray was funded by the US National Institutes of Health (NIH) [U19 CA 148537 for ELucidating Loci Involved in Prostate cancer SuscEptibility (ELLIPSE) project and X01HG007492 to the Center for Inherited Disease Research (CIDR) under contract number HHSN268201200008I]. Additional analytic support was provided by NIH NCI U01 CA188392 (PI: Schumacher).

Aarhus

This study was supported by Innovation Fund Denmark, the Danish Cancer Society and The Velux Foundation (Veluxfonden).
The Danish Cancer Biobank (DCB) is acknowledged for biological material.

AHS
This work was supported by the Intramural Research Program of the NIH, National Cancer Institute, Division of Cancer Epidemiology and Genetics (Z01CP010119).

ATBC
The ATBC Study is supported by the Intramural Research Program of the U.S. National Cancer Institute, National Institutes of Health, and by U.S. Public Health Service contract HHSN261201500005C from the National Cancer Institute, Department of Health and Human Services.

Canary PASS
PASS was supported by Canary Foundation and the National Cancer Institute's Early Detection Research Network )U01 CA086402)

CCI
This work was awarded by Prostate Cancer Canada and is proudly funded by the Movember Foundation - Grant # D2013-36.
The CCI group would like to thank David Murray, Razmik Mirzayans, and April Scott for their contribution to this work.

COH
SLN is partially supported by the Morris and Horowitz Families Endowed Professorship

COSM
COSM is funded by The Swedish Research Council (grant for the Swedish Infrastructure for Medical Population-based Life-course Environmental Research – SIMPLER), the Swedish Cancer Foundation.

CPCS1
Department of Clinical Biochemistry, Herlev and Gentofte Hospital, Copenhagen University Hospital, Herlev Ringvej 75, DK-2730 Herlev, Denmark
We thank participants and staff of the Copenhagen General Population Study for their important contributions.

CPCS2
Department of Clinical Biochemistry, Herlev and Gentofte Hospital, Copenhagen University Hospital, Herlev Ringvej 75, DK-2730 Herlev, Denmark
We thank participants and staff of the Copenhagen General Population Study for their important contributions.

CPS-II
The American Cancer Society funds the creation, maintenance, and updating of the Cancer Prevention Study II cohort.
We thank the CPS-II participants and Study Management Group for their invaluable contributions to this research. We would also like to acknowledge the contribution to this study from central cancer registries supported through the Centers for Disease Control and Prevention National Program of Cancer Registries, and cancer registries supported by the National Cancer Institute Surveillance Epidemiology and End Results program.

EPIC
"The coordination of EPIC was financially supported by the European Commission (DG-SANCO) and the International Agency for Research on Cancer. The national cohorts (that recruited male participants) are supported by Danish Cancer Society (Denmark); German Cancer Aid, German Cancer Research Center (DKFZ), Federal Ministry of Education and Research (BMBF), Deutsche Krebshilfe, Deutsches Krebsforschungszentrum and Federal Ministry of Education and Research (Germany); the Hellenic Health Foundation (Greece); Associazione Italiana per la Ricerca sul Cancro-AIRC-Italy and National Research Council (Italy); Dutch Ministry of Public Health, Welfare and Sports (VWS), Netherlands Cancer Registry (NKR), LK Research Funds, Dutch Prevention Funds, Dutch ZON (Zorg Onderzoek Nederland), World Cancer Research Fund (WCRF), Statistics Netherlands (The Netherlands); Health Research Fund (FIS), PI13/00061 to Granada; , PI13/01162 to EPIC-Murcia), Regional Governments of Andalucía, Asturias, Basque Country, Murcia and Navarra, ISCIII RETIC (RD06/0020) (Spain); Swedish Cancer Society, Swedish Research Council and County Councils of Skåne and Västerbotten (Sweden); Cancer Research UK (14136 to EPIC-Norfolk; C570/A16491 and C8221/A19170 to EPIC-Oxford), Medical Research Council (1000143 to EPIC-Norfolk, MR/M012190/1 to EPIC-Oxford) (United Kingdom). For information on how to submit an application for gaining access to EPIC data and/or biospecimens, please follow the instructions at http://epic.iarc.fr/access/index.php"

ERSPC
This study was supported by the DutchCancerSociety(KWF94-869,98-1657,2002-277,2006-3518, 2010-4800); The Netherlands Organisation for HealthResearch and Development (ZonMW-002822820,22000106,50-50110-98-311, 62300035), The Dutch Cancer Research Foundation(SWOP), and an unconditional grant from Beckman-Coulter-HybritechInc.

ESTHER
The ESTHER study was supported by a grant from the Baden Württemberg Ministry of Science, Research and Arts.
The ESTHER group would like to thank Hartwig Ziegler, Sonja Wolf, Volker Hermann, Heiko Müller, Karina Dieffenbach, Katja Butterbach for valuable contributions to the study.

FHCRC
The FHCRC studies were supported by grants R01-CA080122, R01-CA056678, R01-CA082664, and R01-CA092579, and K05-CA175147 from the US National Cancer Institute, National Institutes of Health, with additional support from the Fred Hutchinson Cancer Research Center (P30-CA015704).
We thank all the individuals who participated in these studies.

Gene-PARE
The Gene-PARE study was supported by grants 1R01CA134444 from the U.S. National Institutes of Health , PC074201 and W81XWH-15-1-0680 from the Prostate Cancer Research Program of the Department of Defense and RSGT-05-200-01-CCE from the American Cancer Society. S.L.K. is supported by 1K07CA187546 from the U.S. National Cancer Institute.

Hamburg-Zagreb

D.L. is supported by the German Cancer Aid (Deutsche Krebshilfe; 70113348).

HPFS
The Health Professionals Follow-up Study was supported by grants UM1CA167552, CA133891, CA141298, and P01CA055075.
We are grateful to the participants and staff of the Physicians’ Health Study and Health Professionals Follow-Up Study for their valuable contributions, as well as the following state cancer registries for their help: AL, AZ, AR, CA, CO, CT, DE, FL, GA, ID, IL, IN, IA, KY, LA, ME, MD, MA, MI, NE, NH, NJ, NY, NC, ND, OH, OK, OR, PA, RI, SC, TN, TX, VA, WA, WY.

IMPACT
The IMPACT study was funded by The Ronald and Rita McAulay Foundation, CR-UK Project grant (C5047/A1232), Cancer Australia, AICR Netherlands A10-0227, Cancer Australia and Cancer Council Tasmania, NIHR, EU Framework 6, Cancer Councils of Victorial and South Australia, Philanthropic donation to Northshore University Health System. We acknowledge support from the National Institute for Health Research (NIHR) to the Biomedical Research Centre at The Institute of Cancer Research and Royal Marsden Foundation NHS Trust.
We acknowledge the IMPACT study steering committee, collaborating centres and participants.

IPO-Porto
The IPO-Porto study was funded by Fundação para a Ciência e a Tecnologia (FCT; UID/DTP/00776/2013 and PTDC/DTP-PIC/1308/2014) and FEDER (POCI-01-0145-FEDER-028245). MC (SFRH/BD/116557/2016) and MPS (SFRH/BD/132441/2017) are research fellows from FCT.
We would like to express our gratitude to all patients and families who have participated in this study.

KULEUVEN
F.C. and S.J. are holders of grants from FWO Vlaanderen (G.0684.12N and G.0830.13N), the Belgian federal government (National Cancer Plan KPC_29_023), and a Concerted Research Action of the KU Leuven (GOA/15/017). TVDB is holder of a doctoral fellowship of the FWO.

LAAPC
This study was funded by grant R01CA84979 (to S.A. Ingles) from the National Cancer Institute, NIH.

Malaysia
The study was funded by the University Malaya High Impact Research Grant (HIR/MOHE/MED/35 to A.R).
We thank all associates in the Urology Unit, University of Malaya, Cancer Research Malaysia (CRM) and the Malaysian Men's Health Initiative (MMHI).

MCC-Spain
"The study was partially funded by the ""Accion Transversal del Cancer"", approved on the Spanish Ministry Council on the 11th October 2007, by the Instituto de Salud Carlos III-FEDER (PI08/1770, PI09/00773-Cantabria, PI11/01889-FEDER, PI12/00265, PI12/01270, PI12/00715, PI15/00069), by the Fundación Marqués de Valdecilla (API 10/09), by the Spanish Association Against Cancer (AECC) Scientific Foundation and by the Catalan Government DURSI grant 2009SGR1489. Samples: Biological samples were stored at the Parc de Salut MAR Biobank (MARBiobanc; Barcelona) which is supported by Instituto de Salud Carlos III FEDER (RD09/0076/00036). Also sample collection was supported by the Xarxa de Bancs de Tumors de Catalunya sponsored by Pla Director d'Oncologia de Catalunya (XBTC). ISGlobal is a member of the CERCA Programme, Generalitat de Catalunya."
We acknowledge the contribution from Esther Gracia-Lavedan in preparing the data. We thank all the subjects who participated in the study and all MCC-Spain collaborators.

MCCS
Melbourne Collaborative Cohort Study (MCCS) cohort recruitment was funded by VicHealth and Cancer Council Victoria. The MCCS was further augmented by Australian National Health and Medical Research Council grants 209057, 396414 and 1074383 and by infrastructure provided by Cancer Council Victoria. Cases and their vital status were ascertained through the Victorian Cancer Registry and the Australian Institute of Health and Welfare, including the National Death Index and the Australian Cancer Database.

MEC
The MEC was supported by NIH grants CA063464, CA054281, CA098758, and CA164973.

MOFFITT
The Moffitt group was supported by the US National Cancer Institute (R01CA128813, PI: J.Y. Park).

Oslo
CONOR was supported by grants from the Nordic Cancer Union, the Swedish Cancer Society (2012/823) and the Swedish Research Council (2014/2269).
The authors wish to acknowledge the services of CONOR, the contributing research centres delivering data to CONOR, and all the study participants.

PCMUS
The PCMUS study was supported by the Bulgarian National Science Fund, Ministry of Education and Science (contract DOO-119/2009; DUNK01/2-2009; DFNI-B01/28/2012) with additional support from the Science Fund of Medical University - Sofia (contract 51/2009; 8I/2009; 28/2010; ).

PHS
The Physicians’ Health Study was supported by grants CA34944, CA40360, CA097193, HL26490 and HL34595.
We are grateful to the participants and staff of the Physicians’ Health Study and Health Professionals Follow-Up Study for their valuable contributions, as well as the following state cancer registries for their help: AL, AZ, AR, CA, CO, CT, DE, FL, GA, ID, IL, IN, IA, KY, LA, ME, MD, MA, MI, NE, NH, NJ, NY, NC, ND, OH, OK, OR, PA, RI, SC, TN, TX, VA, WA, WY.

PLCO
This PLCO study was supported by the Intramural Research Program of the Division of Cancer Epidemiology and Genetics, National Cancer Institute, NIH
The authors thank Drs. Christine Berg and Philip Prorok, Division of Cancer Prevention at the National Cancer Institute, the screening center investigators and staff of the PLCO Cancer Screening Trial for their contributions to the PLCO Cancer Screening Trial. We thank Mr. Thomas Riley, Mr. Craig Williams, Mr. Matthew Moore, and Ms. Shannon Merkle at Information Management Services, Inc., for their management of the data and Ms. Barbara O’Brien and staff at Westat, Inc. for their contributions to the PLCO Cancer Screening Trial. We also thank the PLCO study participants for their contributions to making this study possible.

PRAGGA
PRAGGA was supported by Programa Grupos Emergentes, Cancer Genetics Unit, CHUVI Vigo Hospital, Instituto de Salud Carlos III, Spain.
PRAGGA wishes to thank Victor Muñoz Garzón, Manuel Enguix Castelo, Sara Miranda Ponte, Carmen M Redondo, Manuel Calaza, Francisco Gude Sampedro, Joaquín González-Carreró and the staff of the Department of Pathology and Biobank of University Hospital Complex of Vigo, Instituto de Investigación Sanitaria Galicia Sur (IISGS), SERGAS, Vigo, Spain; Máximo Fraga, José Antúnez and the Biobank of University Hospital Complex of Santiago, Santiago de Compostela, Spain; and Maria Torres, Angel Carracedo and the Galician Foundation of Genomic Medicine.

PROCAP
PROCAP was supported by the Swedish Cancer Foundation (08-708, 09-0677).
We thank and acknowledge all of the participants in the PROCAP study. We thank Carin Cavalli-Björkman and Ami Rönnberg Karlsson for their dedicated work in the collection of data. Michael Broms is acknowledged for his skilful work with the databases. KI Biobank is acknowledged for handling the samples and for DNA extraction. We acknowledge The NPCR steering group: Pär Stattin (chair), Anders Widmark, Stefan Karlsson, Magnus Törnblom, Jan Adolfsson, Anna Bill-Axelson, Ove Andrén, David Robinson, Bill Pettersson, Jonas Hugosson, Jan-Erik Damber, Ola Bratt, Göran Ahlgren, Lars Egevad, and Roy Ehrnström.

PROFILE
We would like to acknowledge the support of the Ronald and Rita McAulay Foundation and Cancer Research UK. We also acknowledge support from the National Institute for Health Research (NIHR) to the Biomedical Research Centre at The Institute of Cancer Research and Royal Marsden Foundation NHS Trust
We acknowledge the Profile study steering committee and participants.

PROGReSS
This research was supported by Spanish Instituto de Salud Carlos III (ISCIII) funding, an initiative of the Spanish Ministry of Economy and Innovation partially supported by European Regional Development FEDER Funds (INT15/00070, INT16/00154, INT17/00133; PI19/01424; PI16/00046; PI13/02030; PI10/00164), and through the Autonomous Government of Galicia (Consolidation and structuring program: IN607B) given to A.Vega.
We would like to thank the patients for their contribution to the study

ProMPT / ProtecT
CRUK, NIHR, MRC, Cambridge Biomedical Research Centre
"ProtecT would like to acknowledge the support of The University of Cambridge, Cancer Research UK. Cancer Research UK grants [C8197/A10123] and [C8197/A10865] supported the genotyping team. We would also like to acknowledge the support of the National Institute for Health Research which funds the Cambridge Bio-medical Research Centre, Cambridge, UK. We would also like to acknowledge the support of the National Cancer Research Prostate Cancer: Mechanisms of Progression and Treatment (PROMPT) collaborative (grant code G0500966/75466) which has funded tissue and urine collections in Cambridge. We are grateful to staff at the Welcome Trust Clinical Research Facility, Addenbrooke’s Clinical Research Centre, Cambridge, UK for their help in conducting the ProtecT study. We also acknowledge the support of the NIHR Cambridge Biomedical Research Centre, the NIHR HTA (ProtecT grant) and the NCRI / MRC (ProMPT grant) for help with the bio-repository. The UK Department of Health funded the ProtecT study through the NIHR Health Technology Assessment Programme (projects 96/20/06, 96/20/99). The ProtecT trial and its linked ProMPT and CAP (The Cluster Randomized Trial of PSA Testing for Prostate Cancer) studies are supported by Department of Health, England; Cancer Research UK grant number C522/A8649, C11043/A4286, C18281/A8145, C18281/A11326, and C18281/A15064, Medical Research Council of England grant number G0500966, ID 75466 and The NCRI, UK. The epidemiological data for ProtecT were generated though funding from the Southwest National Health Service Research and Development. DNA extraction in ProtecT was supported by USA Dept of Defense award W81XWH-04-1-0280, Yorkshire Cancer Research and Cancer Research UK. The authors would like to acknowledge the contribution of all members of the ProtecT study research group.

The views and opinions expressed therein are those of the authors and do not necessarily reflect those of the Department of Health of England. The bio-repository from ProtecT is supported by the NCRI (ProMPT) Prostate Cancer Collaborative and the Cambridge BMRC grant from NIHR.

We acknowledge support from the National Cancer Research Institute (National Institute of Health Research (NIHR) Collaborative Study: “Prostate Cancer: Mechanisms of Progression and Treatment (PROMPT)” (grant G0500966/75466).

We thank the National Institute for Health Research, Hutchison Whampoa Limited, the Human Research Tissue Bank (Addenbrooke’s Hospital), and Cancer Research UK. The authors would like to thank those men with prostate cancer and the subjects who have donated their time and their samples to the Cambridge Biorepository, which were used in this research. We also would like to acknowledge to support of the research staff in S4 who so carefully curated the samples and the follow-up data (Jo Burge, Marie Corcoran, Anne George, and Sara Stearn)."

Richard Martin was supported by a Cancer Research UK Programme Grant (C18281/A19169) and the National Institute for Health Research Bristol Nutrition Biomedical Research Centre based at University Hospitals Bristol NHS Foundation Trust and the University of Bristol.

QLD
The QLD research is supported by The National Health and Medical Research Council (NHMRC) Australia Project Grants [390130, 1009458] and NHMRC Career Development Fellowship, Cancer Australia PdCCRS and Cancer Council Queensland funding to J Batra
The QLD team would like to acknowledge and sincerely thank the urologists, pathologists, data managers and patient participants who have generously and altruistically supported the QLD cohort.

RAPPER
RAPPER is supported by Cancer Research UK [C1094/A11728; C1094/A18504] and Experimental Cancer Medicine Centre funding [C1467/A7286], and the NIHR Manchester Biomedical Research Centre.
The RAPPER group thank Dr. Holly Summersgill for project management.

SEARCH
SEARCH is funded by a programme grant from Cancer Research UK [C490/A10124] and supported by the UK National Institute for Health Research Biomedical Research Centre at the University of Cambridge. The University of Cambridge has received salary support in respect of PP from the NHS in the East of England through the Clinical Academic Reserve.

SFPCS
SFPCS was funded by California Cancer Research Fund grant 99-00527V-10182

SNP_Prostate_Ghent
The study was supported by the National Cancer Plan, financed by the Federal Office of Health and Social Affairs, Belgium.

SPAG
Manchester Cancer Research Centre and MRC – Medical Research Council for PhD studentship work, MUG - Male Uprising in Guernsey, Hope for Guernsey and Wessex Medical Research for research support

STHM2
STHM2 was supported by grants from The Strategic Research Programme on Cancer (StratCan), Karolinska Institutet; the Linné Centre for Breast and Prostate Cancer (CRISP, number 70867901), Karolinska Institutet; The Swedish Research Council (number K2010-70X-20430-04-3) and The Swedish Cancer Society (numbers 11-0287 and 11-0624); Stiftelsen Johanna Hagstrand och Sigfrid Linnérs minne; Swedish Council for Working Life and Social Research (FAS), number 2012-0073
The authors acknowledge the Karolinska University Laboratory, Aleris Medilab, Unilabs and the Regional Prostate Cancer Registry for performing analyses and help to retrieve data. Carin Cavalli–Björkman and Britt-Marie Hune for their enthusiastic work as research nurses. Astrid Björklund for skilful data management. We wish to thank the BBMRI.se biobank facility at Karolinska Institutet for biobank services.

SWOG-PCPT
PCPT is funded by Public Health Service grants U10CA37429 and 5UM1CA182883 from the National Cancer Institute. 
The authors thank the site investigators and staff and, most importantly, the participants from PCPT who donated their time to this trial.

SWOG-SELECT
SELECT is funded by Public Health Service grants U10CA37429 and 5UM1CA182883 from the National Cancer Institute. 
The authors thank the site investigators and staff and, most importantly, the participants from SELECT who donated their time to this trial.

TAMPERE
The Tampere (Finland) study was supported by the Academy of Finland (251074), The Finnish Cancer Organisations, Sigrid Juselius Foundation, and the Competitive Research Funding of the Tampere University Hospital (X51003). The PSA screening samples were collected by the Finnish part of ERSPC (European Study of Screening for Prostate Cancer).
TAMPERE would like to thank Riina Liikanen, Liisa Maeaettaenen and Kirsi Talala for their work on samples and databases.

Toronto
Prostate Cancer Canada Movember Discovery Grant (D2013-17) to RJH; Canadian Cancer Society Research Institute Career Development Award in Cancer Prevention (2013-702108) to RJH

UKGPCS
UKGPCS would also like to thank the following for funding support: The Institute of Cancer Research and The Everyman Campaign, The Prostate Cancer Research Foundation, Prostate Research Campaign UK (now Prostate Action), The Orchid Cancer Appeal, The National Cancer Research Network UK, The National Cancer Research Institute (NCRI) UK. We are grateful for support of NIHR funding to the NIHR Biomedical Research Centre at The Institute of Cancer Research and The Royal Marsden NHS Foundation Trust. UKGPCS should also like to acknowledge the NCRN nurses, data managers and Consultants for their work in the UKGPCS.
UKGPCS would like to thank all urologists and other persons involved in the planning, coordination, and data collection of the study. KM and AL were in part supported from the NIHR Manchester Biomedical Research Centre

ULM
The Ulm group received funds from the German Cancer Aid (Deutsche Krebshilfe).

WUGS
WUGS would like to thank the following for funding support: The Anthony DeNovi Fund, the Donald C. McGraw Foundation, and the St. Louis Men’s Group Against Cancer.
